# Impact of the soil layer on the soil microbial diversity and composition of *Pinus yunnanensis* at the Ailao Mountains subtropical forest

**DOI:** 10.3389/fmicb.2025.1558906

**Published:** 2025-05-29

**Authors:** Haonan Qiao, Qingchao Zeng, Francis Martin, Qi Wang

**Affiliations:** ^1^College of Plant Protection, China Agricultural University, Beijing, China; ^2^Institute of Education, University College London, London, United Kingdom; ^3^Centre INRAE Grand Est-Nancy, Université de Lorraine, INRAE, UMR Interactions Arbres/Microorganismes, Champenoux, France

**Keywords:** co-occurrence pattern, dominant taxa, forest ecosystem, soil microbiome, *Pinus*

## Abstract

Microbial communities residing in forest soils play crucial roles in decomposing organic matter and recycling nutrients, making these ecosystems one of the most diverse habitats on Earth. However, the composition and function of these complex and diverse microbiomes across different soil layers remain largely unknown. In this study, we collected soil samples from various layers and analysed the bacterial and fungal community compositions in experimental forest ecosystems using sequencing techniques. Our findings revealed that the soil layer was the primary factor influencing microbial communities, whereas sampling season had only a marginal effect. The most prevalent bacterial phyla and fungal classes were Acidobacteria, Actinobacteria, Armatimonadetes, Bacteroidetes, Firmicutes, Planctomycetes, Proteobacteria, Verrucomicrobia, and Agaricomycetes. Owing to the heterogeneity of the soil layer environment, we observed distinct patterns in the bacterial and fungal microbiomes across different layers. Moreover, the soil layer affected the network complexity, with fungi exhibiting higher complexity in the upper layer, whereas bacteria showed the opposite trend. Additionally, the dominant bacterial and fungal taxa across all soil layers belonged predominantly to Acidobacteria and Agaricomycetes, respectively. These findings underscore the significance of soil layers in shaping soil microbial communities and highlight the composition and co-occurrence patterns of the microbial communities within these layers.

## Introduction

Soils are one of the largest reservoirs of organic carbon compounds on Earth and serve as sites for most terrestrial ecosystem functions. Consequently, soil processes play a crucial role in the global nutrient cycle (Malhi et al., 1999; Tursová et al., 2012; Baldrian et al., 2012). It is well established that microbes influence all living organisms and are central to many biogeochemical cycles on Earth, driving global carbon and nutrient cycling with direct feedback effects on ecosystem functions and productivity (Baldrian, 2017; Wagg et al., 2019; Bardgett and Putten, 2014; Tao et al., 2020). Forest soils, which cover vast areas of the Northern Hemisphere, accumulate dead plant biomass on the soil surface rather than being removed during harvest (Crowther et al., 2015; Peh et al., 2015). The input of plant litter into forest soils is estimated to be 4–10 t ha^-1^ per year, and, along with contributions from plant roots, it constitutes the primary source of organic matter in these soils. Decomposition of litter is a key process in the formation of temperate forest soils, characterised by a developed organic horizon rich in humic compounds (Šnajdr et al., 2008). However, there is limited understanding of the contribution of the soil microbiome to humic fragments. Therefore, understanding the microbial structure and composition across various soil layers is vital for forest ecosystems.

Current knowledge of soil microbial community diversity and composition is primarily focused on topsoil, where higher concentrations of soil nutrients and organic matter are present (Mortimer and Gui, 2021). However, understanding subsurface soil microbial communities is crucial because of their significant impact on soil formation processes. Several studies have examined the topsoil (upper 20 cm or less of the soil column), which is characterised by more active processes, such as organic matter decomposition, by dividing it into different layers to explore specific stages and reactions (Baldrian, 2017; Leckie et al., 2004). Numerous studies have investigated microbial communities in forest soils, but most have focused on changes in the bacterial or fungal community structures. For instance, Kathryn et al. highlighted changes in bacterial community structures within soil profiles, emphasising the critical role of soil depth (Eilers et al., 2012; Feng et al., 2019; Hao et al., 2021). They also reported that soil fungal community composition varies with soil layers, underscoring the importance of soil organic matter in fungal communities and diversity (Mortimer and Gui, 2021). Moreover, the identity of tree species, which largely determines litter resources, can influence microenvironmental conditions by affecting the soil nutrient content in forests. The composition of soil microorganisms is influenced by various edaphic factors and environmental conditions, with soil pH being the dominant factor in surface soils (Lauber et al., 2009). While soil pH increases with depth, microbial diversity shows an opposite trend (Feng et al., 2019). Additionally, the amounts of soil organic carbon, available potassium, and phosphorus significantly influence the microbial community structure (Bardgett and Putten, 2014; Mortimer and Gui, 2021). Seasonality is also a crucial factor in forest ecosystems because alternating climatic conditions exert decisive control over tree physiology, photosynthesis, and nutrient cycling (Rasche et al., 2010). Oita et al. demonstrated that variations in endophyte communities across tropical forests closely mirror the factors shaping the distribution and diversity of tropical forest trees, highlighting the importance of climate (Oita et al., 2021). Precipitation significantly affects the soil community composition (Mortimer and Gui, 2021; Hao et al., 2021). Furthermore, although the microbiome comprises diverse groups, most researchers have focused only on one group. Previous studies have shown that bacteria and fungi respond differently to environmental changes and impact ecosystem functions, each of which has its own dominant environmental resource conditions (Strickland and Rousk, 2010; de Vries et al., 2018; Jiao et al., 2021; Bahram et al., 2020). Compared to bacteria, fungi may play a unique role by forming hyphal bridges to alleviate nutrient limitations and exhibit strong lignin-degrading abilities, with bacteria and fungi predominating in the early and late stages of litter decomposition, respectively (Strickland and Rousk, 2010; Holland and Coleman, 1987; Boer et al., 2005; Poll et al., 2008). Although ecological processes are ecosystem-specific, they can only be fully understood by considering their overall functions.

Yunnan Province is home to more than half of China’s plant species and has the richest biodiversity among regions at the same latitude worldwide. Therefore, we aimed to deepen our understanding of the soil microbiome in this area and to explore how soil communities vary across different soil layers and seasons. To achieve this, we conducted a field experiment in the Ailao Mountain Nature Reserve, examining the bacterial and fungal communities across 204 samples collected from various soil layers during both the dry and wet seasons. The objectives of this study were to (i) evaluate the relative influence of season and soil layer on microbiome assembly, (ii) explore the differences in soil microbial diversity and community composition across soil layers, and (iii) identify co-occurrence patterns and keystone taxa within different soil layers. Our findings illuminate the assembly patterns and relationship between litter and soil microbiomes, offering valuable insights for the future management and manipulation of soil microbiomes to promote sustainable forestry.

## Materials and methods

### The experimental design, sampling, and DNA extraction

A *P. yunnanensis* field located in Jingdong County, Yunnan Province, China, was selected for this study. The geographic sampling area ranged from 24°29′31.38″ to 24°29′35.86″ in latitude and from 100°59′23″ to 100°59′26.28″ in longitude. *P. yunnanensis* trees were sampled in August 2019, May, and August 2020. The sampled trees had an average circumference of approximately 80 cm to standardise and maximise the reproducibility of the samples. In total, 12 trees (3 plots and 4 trees per plot) were selected and separated by 3–10 m (Supplementary Table S1). The ground was covered with fallen leaves, and humic fragments (HF) formed above the soil at the sampling site. First, we collected the HF using a knife. Each soil core was then divided into two subsamples based on changes in soil appearance (i.e., soil colour). Organic soil (OS, 0–5 cm depth directly below the litter layer) and organic minerals (OM, 5–25 cm depth directly below the litter layer) were collected ~1 m (north and south) from the trunk of each adult tree. In total, we collected (12 trees ×2 directions×3 sampling seasons). However, we obtained only 66 OS and OM samples, except for one direction of the two trees, which collected a mixture of OS and OM samples (Supplementary Figure S1). Soils were homogenised, passed through a 5-mm sieve to remove plant debris and rocks, and divided into two portions. One part of each sample was stored at 4°C to determine physicochemical parameters, while the other part was stored at −80°C for DNA extraction. All soil samples were transported to the laboratory on dry ice and stored at −80°C before DNA extraction. Total DNA was extracted from the soil using a DNeasy PowerSoil kit (Qiagen, Germany) according to the manufacturer’s protocol. The DNA quality and concentration were determined using a NanoDrop ONE spectrophotometre (Thermo Scientific, USA).

### Amplicon generation and sequencing

Bacterial primers 515F (5′-GTGCCAGCMGCCGCGGTAA-3′) and 806R (5′-GGACTACHVGGGTWTCTAAT-3′) towards the bacterial 16S rRNA genes V4 variable region were selected for bacterial community analysis. The fungal primers ITS1F (5′-CTTGGTCATTTAGAGGAAGTAA-3′) and ITS2 (5′-GCTGCGTTCTTCATCGATG-3′), targeting the ITS1 rRNA genes, were used to analyse the fungal community. For amplification, 30 μl reaction mixtures consisted of 15 μl of Phusion® high-fidelity polymerase chain reaction (PCR) Master Mix (New England Biolabs), 0.2 μM of forward and reverse primers, and about 10 ng of template DNA. A Bio-Rad T100 (Bio-Rad Laboratory, CA) instrument was used to perform PCR amplification with the following amplification procedure: 1 min initial denaturation at 98°C, 30 cycles of 10 s at 98°C, 30 s at 50°C, and 30 s at 72°C, with a final 5 min elongation at 72°C. Libraries were generated using the Illumina TruSeq DNA PCR-Free Library Preparation Kit (Illumina, USA) according to the manufacturer’s instructions. Library quality was assessed using a Qubit 2.0 Fluorometer (Thermo Scientific) and an Agilent Bioanalyzer 2100 system. The libraries were sequenced by Novogene Biotech Co., Ltd., on the Illumina NovaSeq platform.

### Bioinformatics analysis

Raw sequences were split according to their unique barcodes, trimmed adaptors, and primer sequences using in-house scripts. The amplicon sequence data were processed using the DADA2 pipeline to process and construct an amplicon sequence variant (ASV) table, as described previously (Zhuang et al., 2020; Callahan et al., 2016). We visualised the quality and filtered reads using the following parameters (maxN = 0, maxEE = c (2,3) for 16S and maxEE = c (3,5) for ITS). In addition, chimeric sequences were removed. Taxonomic assignments for the clustered ASVs were performed using the Ribosomal Database Project (RDP) trainset 16 database for bacteria and the UNITE v2020 database for fungal ASVs. Overall, paired-end sequencing resulted in 14,693,734 and 15,240,840 high-quality reads from 216 samples, which were assembled into 5,646 and 1,170 ASVs for bacteria and fungi, respectively. The phyloseq package was used for downstream analysis of ASVs (Callahan et al., 2016). Non-bacterial ASVs (chloroplasts and mitochondria) were removed. We also filtered ASVs which were not annotated at the phylum level and had low abundance. Finally, we obtained 5,993 and 1,390 ASVs from the bacterial and fungal databases, respectively. All raw sequence data were made available in the NCBI Sequence Read Archive (SRA) database under accession number PRJNA782391.

### Microbiome analysis in R

All statistical analyses were performed on the R platform using the packages. Microbial alpha diversity analysis was performed using the R package phyloseq (Callahan et al., 2016). Beta diversity was estimated according to the Bray-Curtis distance between samples. Differences in community composition were tested using permutational multivariate analysis of variance (PERMANOVA) for Bray-Curtis indices with 1,000 permutations, as implemented in the adonis function of the R vegan package (Zhuang et al., 2020). We used SourceTracker software (v1.0.1) to study the exchange percentages of the soil layers. Source Tracker analysis was constructed by estimating the proportions of HF and OS communities derived from OM soil, HF and OM communities originating from OS soil, and OS and OM communities sourced from HF soil. The percentage value was derived from the statistical average of the results of the SourceTracker (Knights et al., 2011).

To elucidate the microbial interactions in the soil layer or sampling season, microbial association networks for each soil layer or sampling season were created using the ASVs table, where the rows were ASVs and the columns were samples. To reduce network complexity and visual clarity, only ASVs were detected in 90 and 20% of bacterial and fungal samples, respectively. First, a meta-matrix was generated using the R package “SpiecEasi,” which uses LASSO regularisation and cross-validation to detect the most parsimonious network structure in high-dimensional microbial data (Wagg et al., 2019; Kurtz et al., 2015). The lambda ratio was 0.01, and the network was assessed over 20 lambda values for each of the 50 cross-validation permutations to detect the least variable network links using the StARS selection criterion (Liu et al., 2010). The networks were estimated for each permutation using the Glasso graph estimation method. Visualisation of networks and calculation of network topological properties (degree, modularity, etc.) were performed using the interactive platform Gephi (Bastian et al., 2009). ASVs with high degree and closeness centrality values were identified as ‘hub species’ in co-occurrence networks. The cutoffs for hub nodes, which we set as degree > 30 for bacterial and > 20 for fungal and closeness centrality > 0.3 as hub nodes (Xiong et al., 2020).

To identify the microbial taxa responsible for community differentiation among soil layers or sampling seasons, we employed differential abundance analysis, which was performed using the edgeR generalised linear model (GLM) approach (Robinson et al., 2009). Differential ASVs with false discovery rate-corrected *p*-values < 0.05 were identified as indicator ASVs, which were illustrated by ternary plots with the “ggtern” package (Hamilton and Ferry, 2018). In this study, we defined the dominant taxa (ASVs present in at least 80 and 50% of samples for bacterial and fungal microbiomes, respectively, and with a relative abundance ≥ 0.2% for fungal and bacterial microbiomes, respectively). The phylogenetic tree was annotated and visualised using the iTOL software^1^ (Ivica and Peer, 2019). Linear discriminant analysis effect size (LEfSe) was applied [Wilcoxon *p*-value < 0.05, logarithmic LDA score > 1 (fungal microbiome) and 2 (bacterial microbiome)]^2^ to identify biomarkers of the soil layer and sampling season (Segata et al., 2011). A nonparametric statistical test was used to evaluate the taxonomic differences observed between the different soil layers and sampling seasons. All statistical tests performed in this study were considered to be statistically significant at *p* < 0.05.

## Results

### The diversity of the soil microbiome is driven by the soil layer rather than the sampling season

**Figure 1:**
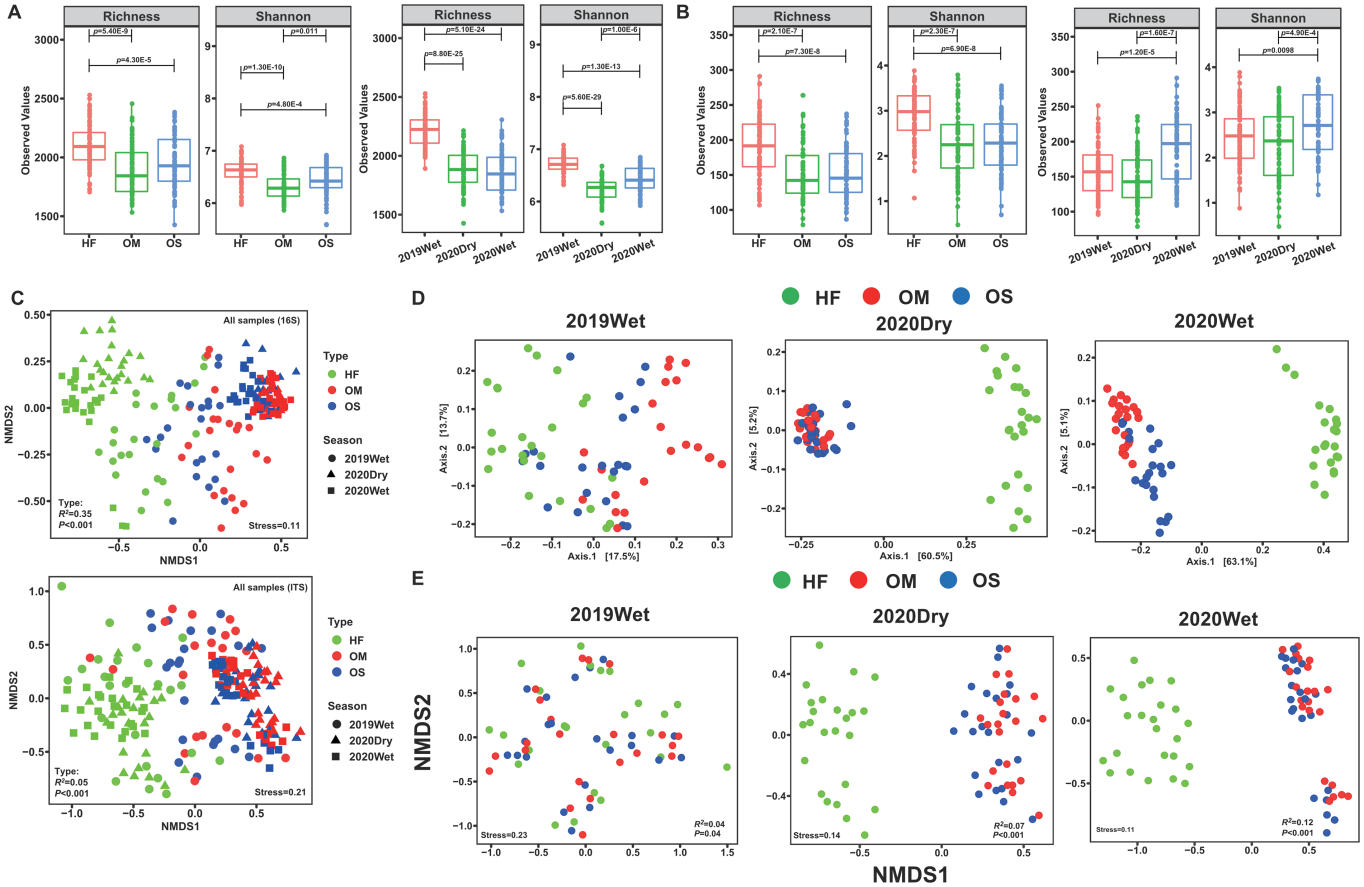
Effects of soil layers on bacterial and fungal communities. Differences in alpha diversity (Observed ASVs and Shannon index) were estimated for the soil layer and sampling season of the bacterial **(A)** and fungal **(B)** communities. **(C)** Non-metric multidimensional scaling (NMDS) ordination of bacterial and fungal community compositions across all samples. Up: bacterial community; down: fungal community. **(D)** Principal coordinate analysis (PCoA) of the bacterial communities during each sampling season. **(E)** NMDS ordination of fungal communities during each sampling season. NMDS and PCoA were based on the Bray-Curtis distance at the ASV level.

According to alpha-diversity indices (Observed ASVs and Shannon index), the bacterial and fungal diversities in HF were significantly higher than those in OS and OM (*p* < 0.01), whereas OS and OM showed similar community diversity (Figures 1A,B). Additionally, 2019Wet displayed significantly higher values for all diversity indices than the 2020Dry and 2020Wet bacterial microbiomes (*p* < 0.01). In contrast, 2020Wet exhibited the highest microbial diversity in the fungal microbiome (*p* < 0.01; Figures 1A,B). For each sampling season, the bacterial and fungal diversity indices of HF were significantly higher than those of OS and OM, except for 2019Wet (*p* < 0.05; Supplementary Figure S2).

To further elucidate the differences in communities between soil layers, we performed non-metric multidimensional scaling (NMDS) ordination analysis which suggested that the overall bacterial and fungal community compositions in the HF were clearly separated from those in the OS and OM samples, which was statistically supported by the PERMANOVA analysis (*R^2^* = 34.73%, *p* < 0.001; *R^2^* = 5.46%, *p* < 0.001) (Figure 1C; Supplementary Table S2). This pattern was repeated by hierarchical clustering of pairwise dissimilarities, which revealed that the HF samples were clearly distinguishable from the OS and OM samples (Supplementary Figure S3). Subsequently, the samples were divided into different sampling seasons and analysed separately. Principal coordinate analysis (PCoA) and NMDS analysis revealed strong clustering of bacterial and fungal microbiomes according to the soil layer, especially for the 2020Dry and 2020Wet samples, whereas the 2019Wet samples were mixed (Figures 1D,E). To statistically support the visual clustering of the microbiome in the above analysis, we performed PERMANOVA and found that the variations in microbial communities were mainly explained by the soil layer (*p* < 0.05, Supplementary Table S3). Within each soil layer, the soil microbiome was significantly shaped by sampling season (21.72 and 5.22% explained the variation in bacterial and fungal communities for HF, 29.88 and 4.35% for OS, and 23.00 and 3.97% for OM), except for the fungal microbiome of OM (*p* < 0.05, Supplementary Figure S4 and Supplementary Table S4). All the results indicated that the soil layer had a certain impact on the soil microbiome.

### Co-occurrence patterns of bacterial and fungal communities in each soil layer

**Figure 2:**
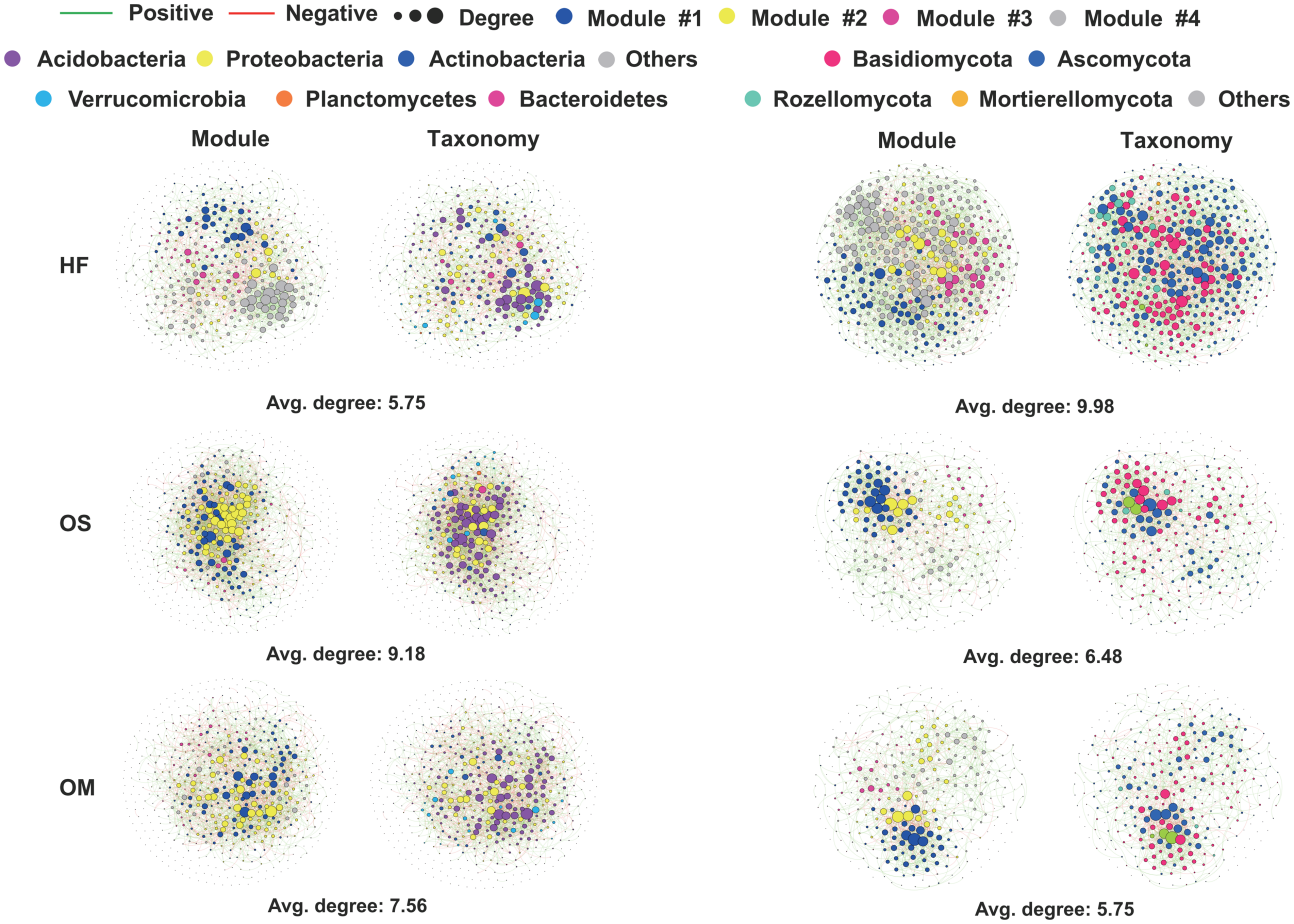
Network visualisation of the interaction architecture in the bacterial and fungal communities of each soil layer. Left: Bacterial networks in each soil layer from all samples. Right: Fungal network of each soil layer in all the samples. Each node colour represents a microbial species at the phylum or class levels. For visual clarity, only ASVs were detected in 90 and 20% of bacterial and fungal samples, respectively.

We further conducted network analysis to illustrate the co-occurrence patterns of bacteria and fungi in each soil layer and calculated their corresponding topological properties. Intriguingly, HF had higher average degrees than OS and OM for the fungal microbiome. The bacterial network complexity decreased from OS (9.18) to OM (7.56), with the lowest network complexity found in the HF soil layer (5.75) (Figure 2 and Table 1). Moreover, we defined ‘hub nodes’ as ASVs with high values of degree (>30 for bacteria and >20 for fungi) and closeness centrality (>0.3) in the network and obtained similar results compared to the network complexity (Table 1). In addition, the composition of the network communities differed between the soil layers. We found more Acidobacteria nodes in the OS (49.9%) and OM (49.4%) than in the HF (36.6%). Meanwhile, HF (20.1 and 33.2%) contained more nodes, which were annotated as Actinobacteria and Proteobacteria, compared with OS (14.6 and 27.8%) and OM (13.7 and 28.2%) samples. In the fungal microbiome, more nodes belonging to Ascomycota were found in the OS and OM of the HF and Basidiomycota (Figure 2). The highest modularity was found in OM for all microbiomes, whereas a higher average path distance was found for bacterial and fungal microbiomes in HF and OM (Table 1).

**Table 1:** Topological properties of co-occurring networks obtained from each soil layer for the bacterial and fungal microbiomes.

	Soil layer	Node	Positive edge	Negative edge	Average degree	Modularity	Average clustering coefficient	Average path distance	Hub node^*^
Bacteria	HF	503	1,147	300	5.753	0.753	0.334	3.735	3
	OS	553	1,613	925	9.179	1.247	0.393	3.317	49
	OM	575	1,394	778	7.555	1.361	0.341	3.452	23
Fungi	HF	465	1,924	397	9.983	0.8	0.236	3.251	29
	OS	337	853	238	6.475	0.95	0.375	3.815	18
	OM	342	783	201	5.754	0.997	0.371	3.96	8

* A hub node is defined as a node with high values of degree (30 for bacteria and 20 for fungi) and closeness centrality (>0.3) in the network.

To gain deeper insight into the effects of seasonality, alpha diversity and microbial networks were assessed. Seasonality has a strong effect on network complexity. The fungal network complexities of HF and OS were higher in the wet season than in the dry season, whereas bacterial network complexity was higher in the dry season than in the wet season. Fungal alpha diversity was lower in the wet season than in the dry season, whereas bacterial alpha diversity was lower in the dry season than in the wet season (Supplementary Figures S5–S7).

### Soil layer shifts the community composition

**Figure 3:**
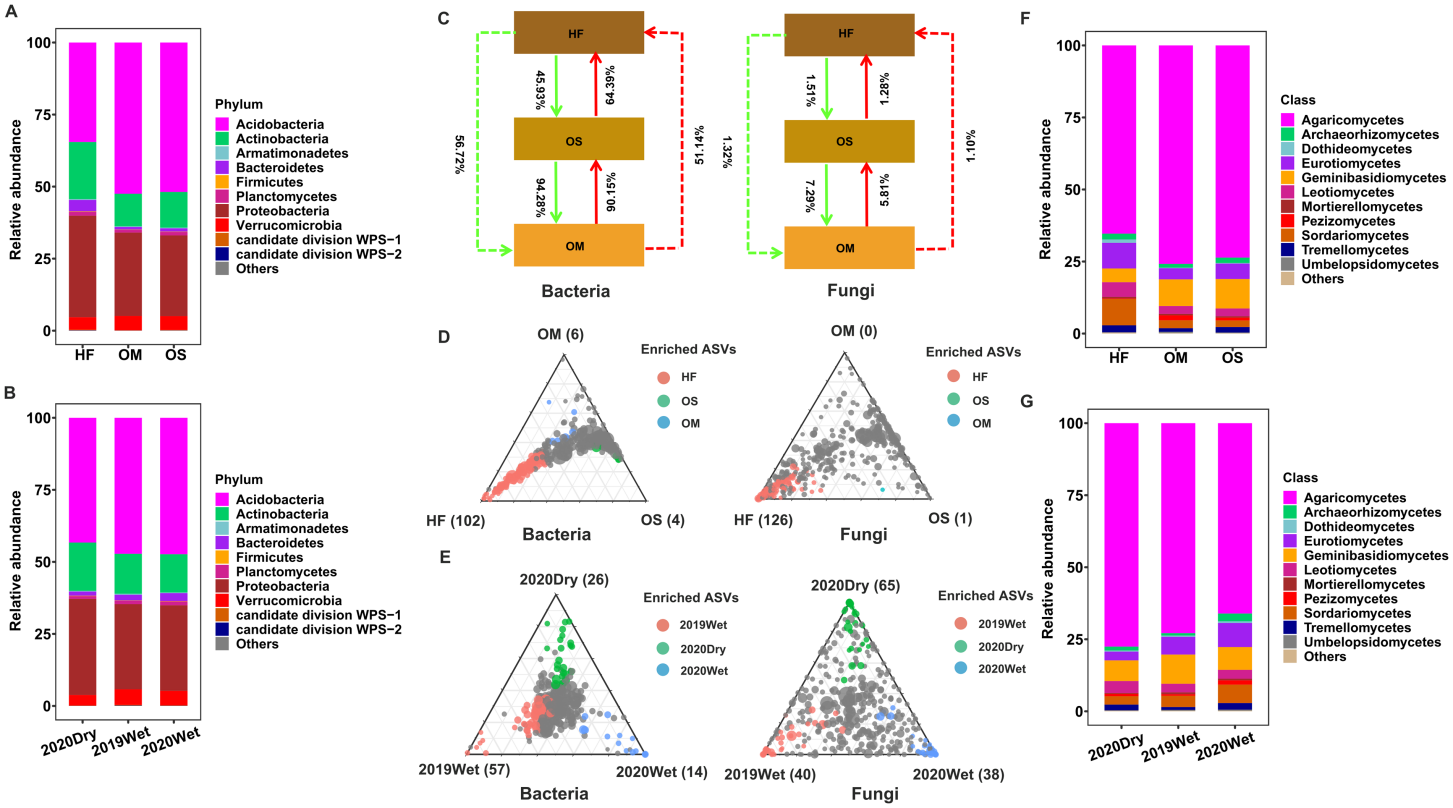
Bacterial and fungal community structures. Taxonomic comparison at the phylum level of the bacterial microbiome of the soil layer **(A)** and the sampling season **(B)**. SourceTracker analysis results for all samples **(C)**. Ternary plots depicting the soil layer **(D)** or sampling season’s **(E)** relative abundance of all ASVs (>0.5%) across bacterial and fungal microbiomes. Each point corresponded to an ASV. Its position represents its relative abundance with respect to each soil layer or sampling season, and its size represents the average across all soil layers or sampling seasons. Coloured circles represent ASVs enriched in each soil layer or sampling season compared to others (red in HF or 2019Wet, green in OS or 2020Dry, and blue in OM or 2020Wet). Taxonomic comparison at the class level of the fungal microbiome of the soil layer **(F)** and sampling season **(G)**. The phyla or classes with less than 0.05% of the average relative abundance are grouped into “Others”.

Bacterial and fungal communities were monitored to investigate the effects of soil layer on the soil microbiome assemblage. The most abundant bacterial phyla in both soil layers were Acidobacteria, Actinobacteria, Armatimonadetes, Bacteroidetes, Firmicutes, Planctomycetes, Proteobacteria, Verrucomicrobia, and the candidate divisions WPS-1 and WPS-2 (average relative abundance > 0.05%). The fungal community composition at the class level in each soil layer is shown in Figure 3. Agaricomycetes were the most dominant class in all soil layers. A similar distribution was observed in different sampling seasons (Figure 3). Importantly, we found substantial differences in bacterial and fungal community structures in each soil layer. The results showed that Actinobacteria, Armatimonadetes, Bacteroidetes, Proteobacteria, and candidate division WPS-2 were significantly increased in HF compared to OS and OM (*p* < 0.01), whereas the abundance of Acidobacteria was reduced (*p* < 2e-16, Supplementary Figure S8). The same effect was observed in the fungal microbiome, and the results showed that the abundance of Dothideomycetes, Eurotiomycetes, Leotiomycetes, Sordariomycetes, and Pezizomycetes and the abundance of Agaricomycetes and Geminibasidiomycetes decreased in HF compared to OS and OM (*p* < 0.01, Supplementary Figure S9). Special attention was paid to the influence of the sampling season on the composition of microbial communities. We found that populations belonging to the Actinobacteria and Proteobacteria phyla were relatively enriched in the dry season, whereas populations of Verrucomicrobia were comparatively depleted compared to the wet season (*p* < 0.01, Supplementary Figure S10). It was noticed that Mortierellomycetes appeared to be more abundant in the fungal microbiome during the wet season (*p* < 0.01; Supplementary Figure S11). It was also observed that the composition of the soil layer and the sampling season process were similar for all samples, with only some discrepancies (Supplementary Figures S12, S13). Overall, the relative abundance analysis indicated that the soil layer had a significant effect on community composition.

Source tracking analysis was conducted to study the exchange proportion of the soil layer. According to the source apportionment results, the exchange proportion of the bacterial microbiome is higher than that of the fungal microbiome. For the bacterial microbiome, the results showed that the majority of bacterial members in OS (90.15%) were derived from OM samples, but rare members were derived from HF samples (45.93%), indicating that there is a clear boundary between HF, OS, and OM (Figure 3). For the fungal microbiome, the exchange proportions of OS and OM were higher than those of OS and HF and also indicated that OS and OM had similar composition patterns. Importantly, we observed that the exchange proportion from the bottom to the top was smaller than that from the HF to OS to OM for the fungal microbiome. In addition, we divided the samples based on the sampling season and calculated the exchange proportion and obtained similar results compared with all the datasets, with some discrepancies (Supplementary Figure S14).

To further elucidate the differences in community turnover between the soil layers, we performed an analysis to identify ASVs that were specifically enriched in the soil layer. A large number of enriched ASVs were observed in HF samples. In contrast, only a small number of ASVs were specifically enriched in OS and OM samples (Figure 3D). The results showed that HF-enriched ASVs were 102 and 126, mostly (more than 85 and 86%) belonging to Acidobacteria, Actinobacteria, Proteobacteria, Agaricomycetes, Eurotiomycetes, Leotiomycetes, and Sordariomycetes, respectively. However, all the OS- and OM-enriched ASVs were annotated as Proteobacteria and Acidobacteria, respectively. This pattern was reproducible when the same analysis was performed in each sampling season (Supplementary Figure S12). Furthermore, to characterise the bacterial and fungal community shifts in different sampling seasons, we also identified sampling season-enriched ASVs in all databases and different soil layers. We found that 2019Wet (57) and 2020Dry (65) had the highest enriched ASVs for bacterial and fungal microbiomes (Figure 3E). However, the pattern was similar for all the databases when we independently performed the same analysis for each soil layer (Supplementary Figure S13).

### Dominant and biomarker taxa of the microbiome in each soil layer

**Figure 4:**
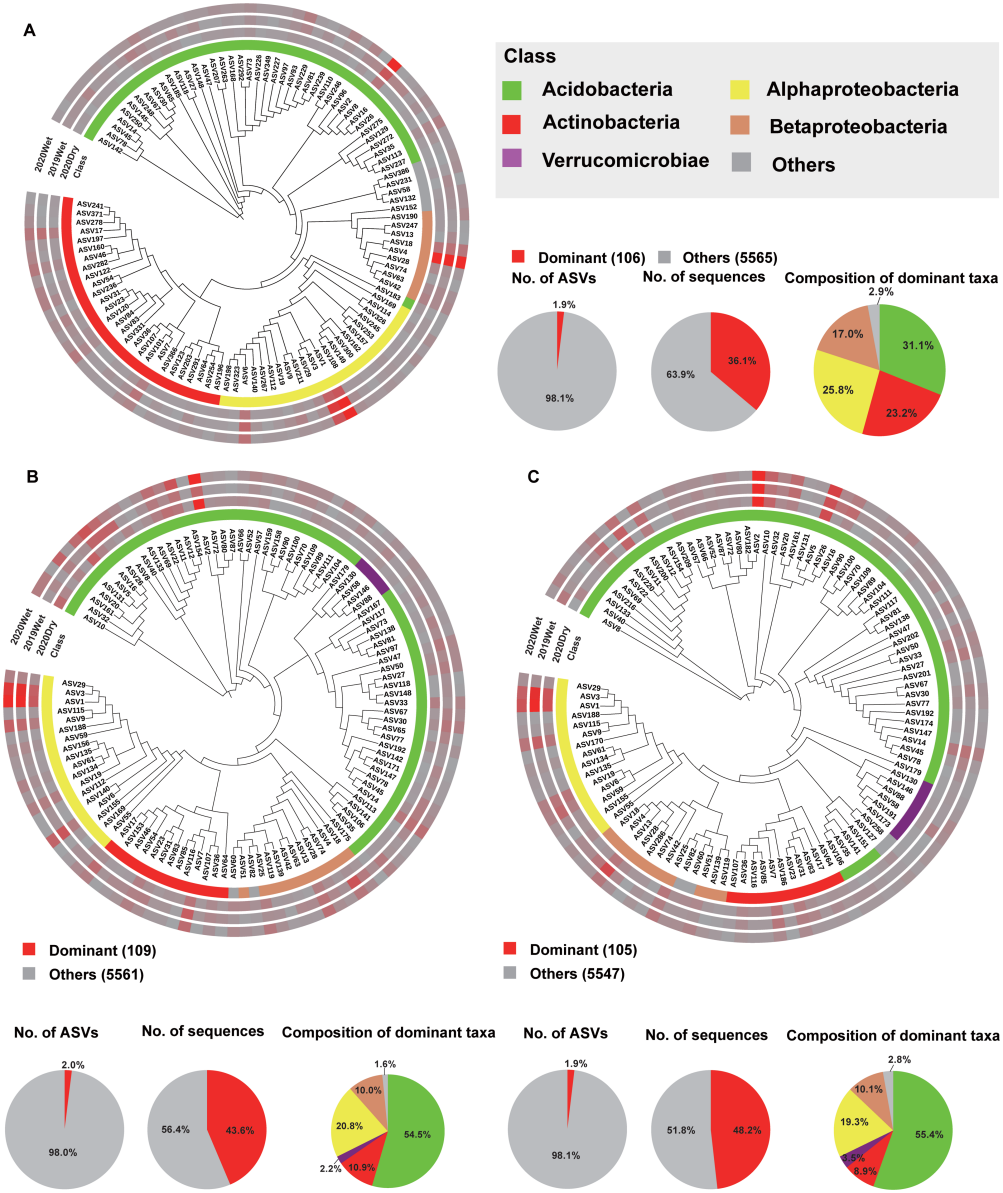
Phylogenetic tree, taxonomic composition, and distribution patterns of soil layer-dominant taxa in the bacterial microbiome. **(A)** Identification of the dominant taxa in HF (*n* = 72). **(B)** Identification of dominant taxa in the OS (*n* = 66). **(C)** Identification of the dominant OM taxa (*n* = 66). Dominant taxa were defined as ASVs present in >80% of all the samples, with an average relative abundance of ≥0.2%. Low-abundance classes with <2% of the total sequences of dominant taxa across different soil layers are grouped into ‘Others’.

**Figure 5:**
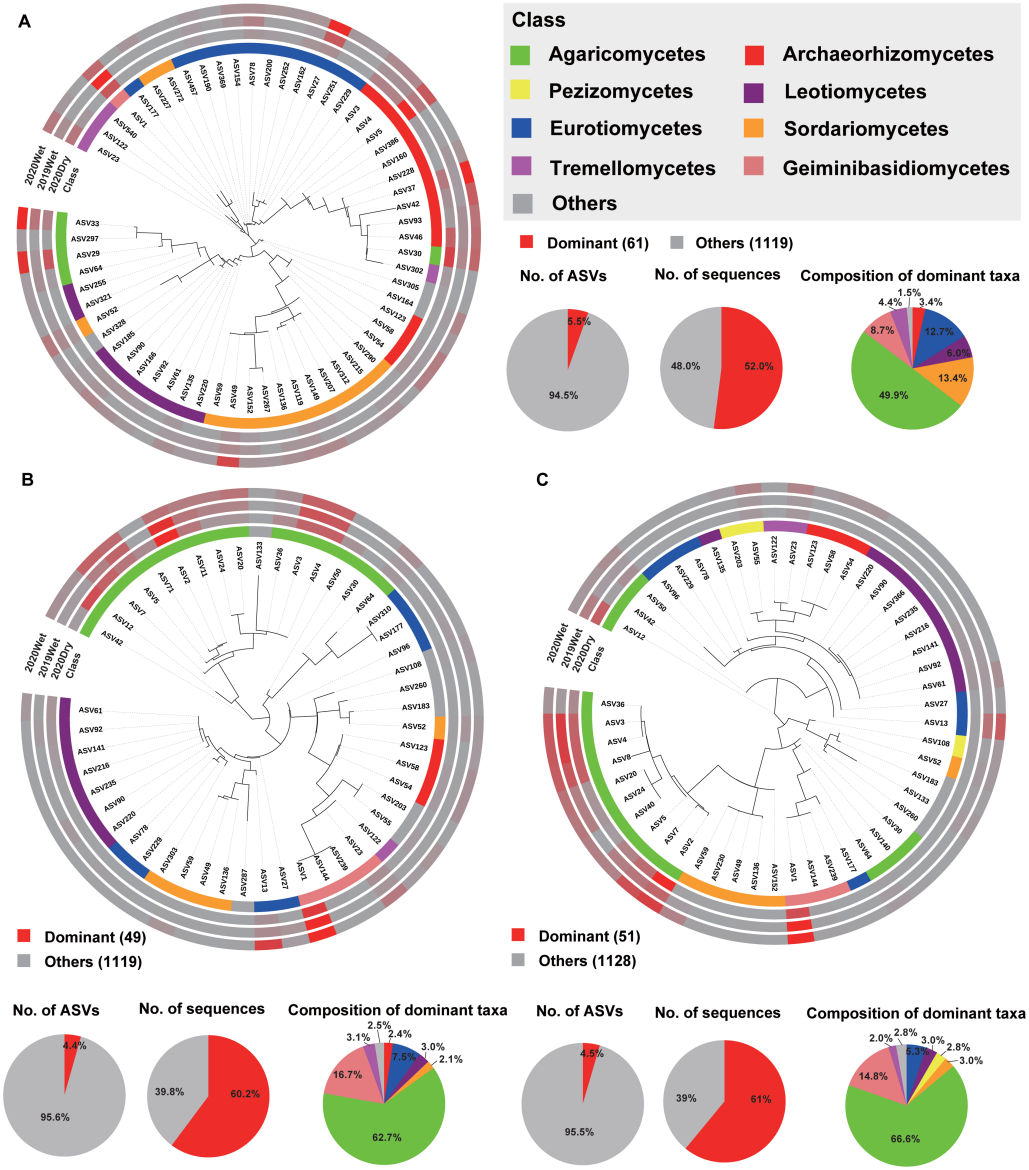
Phylogenetic tree, taxonomic composition, and distribution patterns of each soil layer’s dominant taxa in the fungal microbiome. **(A)** Identification of dominant taxa in HF (*n* = 72). **(B)** Identification of dominant taxa in OS (*n* = 66). **(C)** Identification of the dominant OM taxa (*n* = 66). Dominant taxa were defined as ASVs present in > 50% of all samples, with an average relative abundance of ≥0.2%. Low-abundance classes with <2% of the total sequences of dominant taxa across different soil layers are grouped into ‘Others’.

To further characterise the soil layer effect on the microbiome, we surveyed the dominant taxa (ASVs present in at least 80% (bacteria) and 50% (fungi) of the samples, with a relative abundance >0.2%) and biomarker taxa for each soil layer. Among all ASVs obtained from each soil layer, only 106 (1.9%), 109 (2.0%), and 105 (1.9%) ASVs were identified as the dominant taxa for the HF, OS, and OM bacterial microbiomes, respectively. These ASVs accounted for 42.6% (36.1, 43.6, and 48.2%, respectively) of the total sequences in each soil layer. For all soil layers, these dominant ASVs were mainly Acidobacteria, with a relative abundance of 31.1–55.4% within each soil layer. In total, 34 dominant taxa were shared by the soil layer, 13 of which were annotated as Acidobacteria and 12 of which were annotated as Proteobacteria (Figures 4A–C; Supplementary Figure S15A; Supplementary Table S5). We found that 61 (5.5%), 49 (4.4%), and 51 (4.5%) ASVs were the dominant taxa in HF, OS, and OM fungal microbiomes, respectively. These ASVs accounted for 57.7% (52.0, 60.2, and 61.0%, respectively) of the total sequences in each soil layer. For the fungal microbiome, the dominant ASVs were mainly Agaricomycetes, with a relative abundance of 49.9–66.6% within each soil layer. In total, 24 dominant taxa were shared by the different soil layers, six of which were annotated as Agaricomycetes (Figure 5; Supplementary Figure S15B; Supplementary Table S6). We also focused on the sampling season effect, and the results were similar to those of the analysis of the soil layers. The results showed that 109 (1.9%), 113 (2.2%), and 126 (2.3%) ASVs were the dominant taxa for 2019Wet, 2020Dry, and 2020Wet of the bacterial microbiome, respectively. These ASVs accounted for 39.9% (34.6, 45.5, and 39.5%, respectively) of the total sequences in each soil layer. For all soil layers, these dominant ASVs were mainly Acidobacteria, with a relative abundance of 44.2–50.9% within each soil layer. In total, 75 dominant taxa were shared by sampling season, 37 of which were annotated as Acidobacteria, and the remaining were annotated as Actinobacteria, Proteobacteria, and Verrucomicrobia at the phylum level (Supplementary Figure S15; Supplementary Table S7). Moreover, 60 (5%), 62 (7%), and 75 (7%) ASVs were identified as the dominant taxa in the 2019Wet, 2020Dry, and 2020Wet fungal microbiomes, respectively. These ASVs accounted for 50.8% (22.9, 62.9, and 67.6%, respectively) of the total sequences in each soil layer. For the fungal microbiome, these dominant ASVs were mainly Agaricomycetes in 2020Dry and 2020Wet, with a relative abundance of 59.5–72.7% within each soil layer, whereas it was mainly Geminibasidiomycetes in 2019Wet. In total, 33 dominant taxa were shared among the different soil layers (Supplementary Figure S17; Supplementary Table S8).

Analysis of the LDA effect size (LEfSe) revealed that Acidobacteria in HF, while uncultured bacteria belonging to the class Acidobacteria in OS and OM, were the most significant biomarker taxa. In the fungal microbiome, Eurotiomycetes and Pezizomycetes in HF and OM were the most significant biomarker taxa (Supplementary Figures S15C,D). Furthermore, we identified the dominant taxa in different sampling seasons. The results showed that Actinobacteria in 2020Dry, unidentified bacteria belonging to the phylum Actinobacteria in 2019Wet, and Bacteroidetes in 2020Wet were the most significant biomarker taxa in the bacterial microbiome. Tremellales in 2020Dry, *Lactarius* in 2019Wet, and Sordariomycetes in 2020Wet were the most significant biomarker taxa of the fungal microbiome (Supplementary Figures S18C,D).

## Discussion

A survey examining the microbial community composition across different soil layers in a *P. yunnanensis* plantation during the dry and wet seasons revealed that the soil layers significantly shaped microbiomes, whereas the influence of the sampling season was minimal. Additionally, our findings indicate that soil layers have a pronounced impact on network complexity. These results underscore the role of soil layers in influencing the microbiome assembly. We also identified microbial community composition, exchange proportion, and dominant taxa in each soil layer. These insights substantially enhance our current understanding of microbial community assembly across different soil layers in forest ecosystems and offer a comprehensive perspective on soil-layer biogeography.

### Soil layer is the main factor to influence the microbiome assembly

The microbiome plays a crucial role in soil ecosystems by affecting processes such as soil formation, fertility, nutrient turnover, and carbon storage (Naylor et al., 2022). Our findings revealed that the bacterial and fungal diversity was significantly higher in HF than in OS and OM (Figure 1). This pattern of decreasing microbial alpha diversity aligns with previous studies on paddy and forest soils, which have shown that diversity generally diminishes with increasing soil depth (Ren et al., 2015; Yta et al., 2018; Levy-Booth et al., 2016). Surface-dwelling microbes exhibit reduced survival in subsurface soils (Eilers et al., 2012). This may also be attributed to the decline in the availability of various resources and oxygen with soil depth as well as the greater heterogeneity of nutrient sources in the upper layers (Wu et al., 2019; Fierer et al., 2007). Typically, the transition zone for the microbial community ranges from 10 to 25 cm in natural forest soils (Eilers et al., 2012). In this study, we focused on the microbiome composition of the topsoil (~25 cm from the soil surface). However, we also observed that the alpha diversity of OS was higher than that of OM, particularly for the bacterial microbiome (Figure 1). Additionally, our results indicated that microbiome assembly was primarily determined by the soil layer, and the exchange proportion between OS and OM was significantly higher than that in the other layers (Figures 1, 3).

Environmental conditions such as soil features and precipitation can influence the soil microbial community. The physicochemical characteristics of the soil, which vary with depth, show significant positive correlations (SOC and total N) and negative correlations (bulk density and pH) with microbial community diversity (Mortimer and Gui, 2021; Feng et al., 2019; Jiao et al., 2018). The soil pH and SOC at different depths may exert a stronger influence on community structure. Our findings indicate that the pH of OM is higher than that of OS; however, the organic matter content of OS was significantly greater than that of OM (*p* < 0.01, data not shown). Previous studies have confirmed that the soil characteristics of the HF layer differ substantially from those of the OS and OM layers (Seuradge et al., 2017; López-Mondéjar et al., 2015). Numerous studies have emphasised the impact of soil moisture and rainfall frequency on the structure of soil microbiome communities (Chemidlin Prevost-Boure et al., 2011; Cregger et al., 2012). Although the sampling season significantly affected the microbiome, its impact was minor compared with that of the soil layer. Additionally, we observed a higher microbial diversity during the wet season, particularly in the fungal microbiome (Figure 1; Supplementary Figure S2). Lower precipitation can decrease bacterial diversity in tropical forests (He et al., 2017). At the Kanawha site, where precipitation is greater than at the other two sites, the topsoil exhibits a more distinct microbial community (Hao et al., 2021). Notably, water addition increases microbial biomass C and N as well as bacterial and fungal abundance (Huang et al., 2015).

### Similarities and differences between the soil layers of community composition

This vertical stratification is marked by a decline in both the content and quality of organic matter as soil depth increases, accompanied by shifts in extracellular enzyme activity and microbial community composition (Šnajdr et al., 2008; Poll et al., 2008; Zechmeister, 2015; Craig et al., 2022; Trivedi et al., 2013). In line with previous findings, we demonstrated that Acidobacteria and Proteobacteria, along with, to a lesser extent, Actinobacteria, Bacteroidetes, Planctomycetes, and Verrucomicrobia, dominated the soil bacterial assemblages, whereas Agaricomycetes was the most prevalent class in the fungal microbiome (Figure 3) (Hao et al., 2021; Dang et al., 2018). Changes in the soil microbiome with depth were evident in abundance patterns. It was observed that Actinobacteria, Armatimonadetes, Bacteroidetes, and Proteobacteria were significantly more abundant in the HF layer than in the OS and OM layers, whereas Acidobacteria was significantly less abundant (Supplementary Figure S8). The availability of carbon to soil microorganisms influences microbial communities. Previous studies have shown that as C mineralisation rates increase, the abundance of Acidobacteria decreases, whereas the abundances of Proteobacteria and Bacteroidetes increase significantly (Fierer et al., 2007; Ko et al., 2017). Šnajdr et al. (2008) reported a substantial reduction in soil respiration with depth, indicating that organic matter in deeper horizons was more recalcitrant (Šnajdr et al., 2008; López-Mondéjar et al., 2015). The physiological and metabolic traits of microbial taxa may help explain these depth-related abundance trends, as some members can utilise more recalcitrant C sources and tolerate low-nutrient conditions. For instance, Proteobacteria and Bacteroidetes are less prevalent in deep soil (Naylor et al., 2022). Additionally, the relative abundances of Actinobacteria and Proteobacteria during the dry season were significantly higher than those during the wet season for the bacterial microbiome (Supplementary Figure S10). This trend during the dry season aligns with previous reports showing that Actinobacteria and Proteobacteria are recruited by plants to cope with drought stress (Xu et al., 2018).

The predominance of two fungal groups, Ascomycota and Basidiomycota, observed in our study aligns with the findings of other studies (Dang et al., 2018). This dominance in soils may be attributed to their capacity to break down the complex lignocellulose components in plant detritus. We noted that community abundance varied across different soil layers. Specifically, the abundance of Dothideomycetes, Eurotiomycetes, Leotiomycetes, Sordariomycetes, and Pezizomycetes was significantly higher in HF, while Agaricomycetes and Geminibasidiomycetes were more prevalent in OS and OM (Supplementary Figure S9). Notably, all taxa enriched in HF belonged to Ascomycota, whereas those in OS and OM were classified as Basidiomycota. This pattern likely resulted from the influence of soil organic matter on the fungal community composition. Previous findings strongly suggest that variations in soil organic matter between the two soil horizons shape soil fungal communities (Mortimer and Gui, 2021). Ascomycota plays a crucial role in degrading organic matter, affecting soil fertility, and its presence diminishes with depth, as its members are primarily saprotrophic and thus concentrated at surfaces where plant litter is abundant (Naylor et al., 2022). Additionally, Basidiomycota is recognised as a dominant and widely distributed fungus that utilises a broad range of carbon sources, giving it greater prominence in deeper soil layers with more recalcitrant carbon sources (Bastida et al., 2019; Fontaine et al., 2011; López-Mondéjar et al., 2018; Mašínová et al., 2017). Collectively, these results demonstrated that each soil layer hosts a specific microbiome to adapt to a heterogeneous decomposition environment. We also discovered that precipitation plays a vital role in determining fungal community structure, which is consistent with previous studies (Supplementary Figure S11) (Mortimer and Gui, 2021). Water is essential for SOM (soil organic matter) absorption and the metabolic activity of soil organic matter.

### The dominant taxa and hub nodes for each soil layer

The dominant taxa were identified as potential keystone taxa, which play a crucial ecological role in microbiome assembly and ecosystem function (Delgado-Baquerizo et al., 2018; Banerjee and Schlaeppi, 2018). In line with published studies, we observed that less than 2% of the bacterial and 5% of the fungal phylotypes consistently constituted approximately 50% of the microbial community. This suggests that only a few microbial taxa dominate across different soil layers despite the presence of a highly diverse microbial population (Delgado-Baquerizo et al., 2018). Our findings revealed that the dominant bacterial and fungal taxa were Acidobacteria and Agaricomycetes, respectively. Acidobacteria exhibit high metabolic versatility, allowing them to adapt well to resource limitations and decompose complex carbon substrates derived from the recalcitrant soil organic matter pool (Delgado-Baquerizo et al., 2017; Maestre et al., 2015). The role of Acidobacteria in decomposing materials and mobilising nutrients is vital in forest ecosystems that contain vast reservoirs of dead trees and litter. Similarly, Agaricomycetes are essential for nutrient cycling, including carbon sequestration in forest soils (Philippot et al., 2013). Dominant taxa may be key microorganisms that respond to environmental changes and exert significant control over the function and composition of the microbiome (Banerjee and Schlaeppi, 2018).

Interaction networks can enhance ecological theories regarding cooperation and competition among soil community members (Naylor et al., 2022). Notably, the complexity of fungal networks decreased with increasing soil depth, peaking in the HF layer, whereas the bacterial complexity was minimal in the same layer (Figure 2; Table 1). Research has indicated that fungi quantitatively dominate bacteria in decomposing litter material, although the significance of bacteria increases with increasing soil depth (Baldrian et al., 2012; Bååth and Anderson, 2003). Despite evidence that both fungal and bacterial decomposers can degrade complex substrates in forest soils, fungi exhibit a greater capability and can subsequently feed on bacterial biomass (Bastida et al., 2019; López-Mondéjar et al., 2018). This distinction may explain the different roles of fungi and bacteria in the specific soil layers. Fungi are predominantly aerobic and favour plant polysaccharides as substrates (Koranda et al., 2014). Bacteria, on the other hand, are more prevalent in alkaline soils compared to fungi. Additionally, fungal-to-bacterial ratios showed a significant positive correlation with C/N ratios, which decreased with depth (Rousk et al., 2010; Fierer et al., 2009; Tripathi et al., 2019). Within the fungal microbiome, more nodes associated with Ascomycota were identified in HF, whereas Basidiomycota were more prevalent in OS and OM (Figure 2). The distribution patterns of hub nodes varied across soil layers, with different microbes emerging as key hubs in various layers. Ascomycota, which are abundant in the hub nodes of the HF, have been reported to play a crucial role in degrading organic matter and potentially influencing soil fertility.

## Data Availability

All raw sequence data were made available in the NCBI Sequence Read Archive (SAR) database under the accession number PRJNA782391.

## Footnotes

^1^ https://itol.embl.de/

^2^ http://huttenhower.sph.harvard.edu/galaxy/

## Supplementary material

SUPPLEMENTARY FIGURE S1 Experimental design and layout of samples in different seasons. The diagram illustrates the different soil layers and different clusters of sampling and sample details for the different sampling seasons.

SUPPLEMENTARY FIGURE S2 Comparative analysis of the alpha diversity of bacterial and fungal communities in each soil layer during the sampling season.

SUPPLEMENTARY FIGURE S3 Hierarchical clustering based on Jaccard and Euclidean distances of ASVs from bacterial and fungal samples. Samples were clustered according to ward.D2 and the complete method for bacterial and fungal microbiomes. **(A)** Bacterial and **(B)** fungal samples.

SUPPLEMENTARY FIGURE S4 NMDS ordinations based on Bray-Curtis distances of bacterial and fungal communities for each soil layer.

SUPPLEMENTARY FIGURE S5 The sampling season affects the bacterial and fungal diversity and the network complexity of HF. Comparative analysis of alpha diversity of bacterial **(A)** and fungal **(B)** communities during the sampling season. **(C)** Bacterial (left) and fungal (right) co-occurrence networks during sampling season. For visual clarity, only ASVs that were detected in 95% and 20% of all bacterial and fungal samples within the sampling season are illustrated.

SUPPLEMENTARY FIGURE S6 The sampling season affects the bacterial and fungal diversity and network complexity of the OS. Comparative analysis of the alpha diversity of bacterial **(A)** and fungal **(B)** community from sampling season. **(C)** Bacterial (left) and fungal (right) co-occurrence networks along the sampling season. For visual clarity, only ASVs that were detected to be present in 95% and 20% of all bacterial and fungal samples within sampling season are illustrated.

SUPPLEMENTARY FIGURE S7 Seasonality affects the bacterial and fungal diversity and network complexity of OM. Comparative analysis of the alpha diversity of bacterial **(A)** and fungal **(B)** community from sampling season. **(C)** Bacterial (left) and fungal (right) co-occurrence networks along the sampling season. For visual clarity, only ASVs that were detected to be present in 95% and 20% of all bacterial and fungal samples within sampling season are illustrated.

SUPPLEMENTARY FIGURE S8 The relative abundance of different bacterial phyla varied among the different soil layers. Statistical analysis of the data was performed using ANOVA. Different letters indicate significant differences.

SUPPLEMENTARY FIGURE S9 The relative abundances of different fungal classes varied among the different soil layers. Statistical analysis of the data was performed using ANOVA analysis. Different letters indicate a significant difference.

SUPPLEMENTARY FIGURE S10 The relative abundance of different bacterial phyla varied among the sampling seasons. Statistical analysis of the data was performed using ANOVA analysis. Different letters indicate a significant difference.

SUPPLEMENTARY FIGURE S11 The relative abundances of different fungal classes varied among the sampling seasons. Statistical analysis of the data was performed using ANOVA analysis. Different letters indicate a significant difference.

SUPPLEMENTARY FIGURE S12 Community composition in each sampling season for the soil layer. The relative abundance of the most abundant bacterial and fungal microbiomes (**A,C:** 2019Wet, **D,F:** 2020Dry, **G,I:** 2020Wet) and the bacterial and fungal-enriched ASVs among each soil layer in different sampling seasons (**B:** 2019Wet, **E:** 2020Dry, **H:** 2020Wet). The phyla with less than 0.5% of the average relative abundance are grouped into “Other”. Ternary plots depicting the relative abundance of all ASVs (>0.5%) across bacterial and fungal microbiomes. Each point corresponded to an ASVs. Its position represents its relative abundance with respect to each soil layer and its size represents the average across all soil layers. Coloured circles represent ASVs enriched in one soil layer compared to the others (red in HF, green in OS, and blue in OM).

SUPPLEMENTARY FIGURE S13 Community composition of each soil layer during sampling season. The relative abundance of the most abundant bacterial and fungal microbiomes (**A,C**: HF, **D,F**: OS, **G,I**: OM) and the bacterial and fungal-enriched ASVs among different sampling seasons in different soil layers (**B**: HF, **E**: OS, and **H**: OM). The phyla with less than 0.05% of the average relative abundance are grouped into “Other”. Ternary plots depicting different sampling seasons relative abundance of all ASVs (>0.5%) across the bacterial and fungal microbiome. Each point corresponds to an ASVs. Its position represents its relative abundance with respect to each sampling season and its size represents the average across all sampling seasons. Coloured circles represent ASVs enriched in one sampling season compared to the others (red in 2020Wet, green in 2019Wet, and blue in 2020Dry).

SUPPLEMENTARY FIGURE S14 Source model showing potential exchange based on all samples from different sampling seasons. **(A)** 2019Wet, **(B)** 2020Dry, and **(C)** 2020Wet.

SUPPLEMENTARY FIGURE S15 Specific and shared dominant taxa and biomarker taxa of the bacterial and fungal microbiomes in each soil layer. Specific and shared dominant taxa among different soil layers for bacterial **(A)** and fungal **(B)** microbiomes. LEfSe identified biomarker taxa associated with each soil layer. Only the top 10 most specific biomarker taxa are shown for bacterial **(C)** and fungal microbiomes **(D)**.

SUPPLEMENTARY FIGURE S16 Phylogenetic tree, taxonomic composition, and distribution patterns of sampling season dominant taxa from the bacterial microbiome. **(A)** Identification of the dominant taxa in 2019Wet (*n* = 68). **(B)** Identification of the dominant taxa in 2020Dry (*n* = 68). **(C)** Identification of the dominant taxa in 2020Wet (*n* = 68). The dominant taxa were defined as ASVs present in more than 80% of all samples and with an average relative abundance ≥0.2%. Low abundance classed with <2% of the total sequences of dominant taxa across different soil layers are grouped into ‘Others’.

SUPPLEMENTARY FIGURE S17 Phylogenetic tree, taxonomic composition, and distribution patterns of sampling season-dominant taxa from the fungal microbiome. **(A)** Identification of dominant taxa for 2019Wet (*n* = 68). **(B)** Identification of dominant taxa for 2020Dry (*n* = 68). **(C)** Identification of dominant taxa for 2020Wet (*n* = 68). The dominant taxa were defined as ASVs present in more than 50% of all samples and with an average relative abundance ≥0.2%. Low abundance, classified as <2% of the total sequences of dominant taxa across each soil layer, was grouped into ‘Others’.

SUPPLEMENTARY FIGURE S18 Specific and shared biomarker taxa of bacterial and fungal microbiomes during the sampling season. Specific and shared dominant taxa among the sampling seasons for bacterial **(A)** and fungal **(B)** microbiomes. LEfSe identified biomarker taxa associated with each sampling season. Only the top 10 most specific biomarker taxonomies were shown for bacterial **(C)** and fungal microbiome **(D)**.

SUPPLEMENTARY TABLE S1 Location of the collected *P. yunnanensis* in the Ailao mountain subtropical forest.

SUPPLEMENTARY TABLE S2 Effects of soil layer and sampling season on bacterial and fungal communities based on PERMANOVA.

SUPPLEMENTARY TABLE S3 Effects of soil layer on bacterial and fungal communities during the sampling season based on PERMANOVA.

SUPPLEMENTARY TABLE S4 Effects of season on the bacterial and fungal communities in each soil layer based on PERMANOVA analysis.

SUPPLEMENTARY TABLE S5 Shared dominant taxa in each soil layer of the bacterial microbiome.

SUPPLEMENTARY TABLE S6 Shared dominant taxa in each soil layer of the fungal microbiome.

SUPPLEMENTARY TABLE S7 Shared dominant taxa in the sampling season of the bacterial microbiome.

SUPPLEMENTARY TABLE S8 Shared dominant taxa of the fungal microbiome in the sampling season.
