# Supplementary material for: Impact of the soil layer on the soil microbial diversity and composition of Pinus yunnanensis at the Ailao Mountains subtropical forest
Source: Front Microbiol. 2025 May 29;16:1558906. doi: 10.3389/fmicb.2025.1558906 (PMC12159057; doi:10.3389/fmicb.2025.1558906)

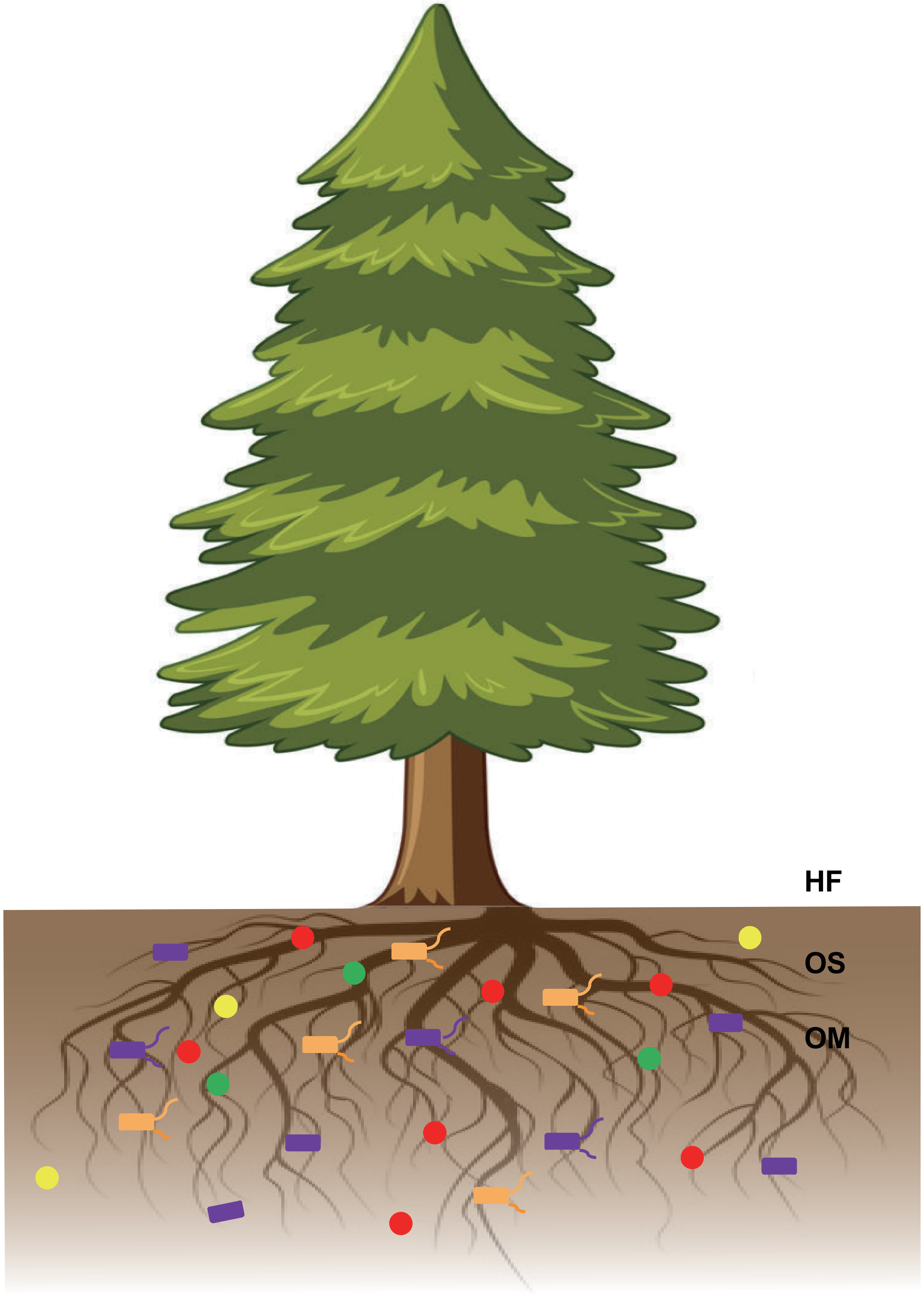

	1. Wet in 2019 (n=68)		
Different soil layer	Cluster1	Cluster2	Cluster3
HF	T1-T4 (n=8)	T5-T8 (n=8)	T9-T12 (n=8)
OS	T1-T4 (n=8)	T5-T8 (n=6)	T9-T12 (n=8)
OM	T1-T4 (n=8)	T5-T8 (n=6)	T9-T12 (n=8)

	2. Dry in 2020 (n=68)		
Different soil layer	Cluster1	Cluster2	Cluster3
HF	T1-T4 (n=8)	T5-T8 (n=8)	T9-T12 (n=8)
OS	T1-T4 (n=8)	T5-T8 (n=6)	T9-T12 (n=8)
OM	T1-T4 (n=8)	T5-T8 (n=6)	T9-T12 (n=8)

	3. Wet in 2020 (n=68)		
Different soil layer	Cluster1	Cluster2	Cluster3
HF	T1-T4 (n=8)	T5-T8 (n=8)	T9-T12 (n=8)
OS	T1-T4 (n=8)	T5-T8 (n=6)	T9-T12 (n=8)
OM	T1-T4 (n=8)	T5-T8 (n=6)	T9-T12 (n=8)

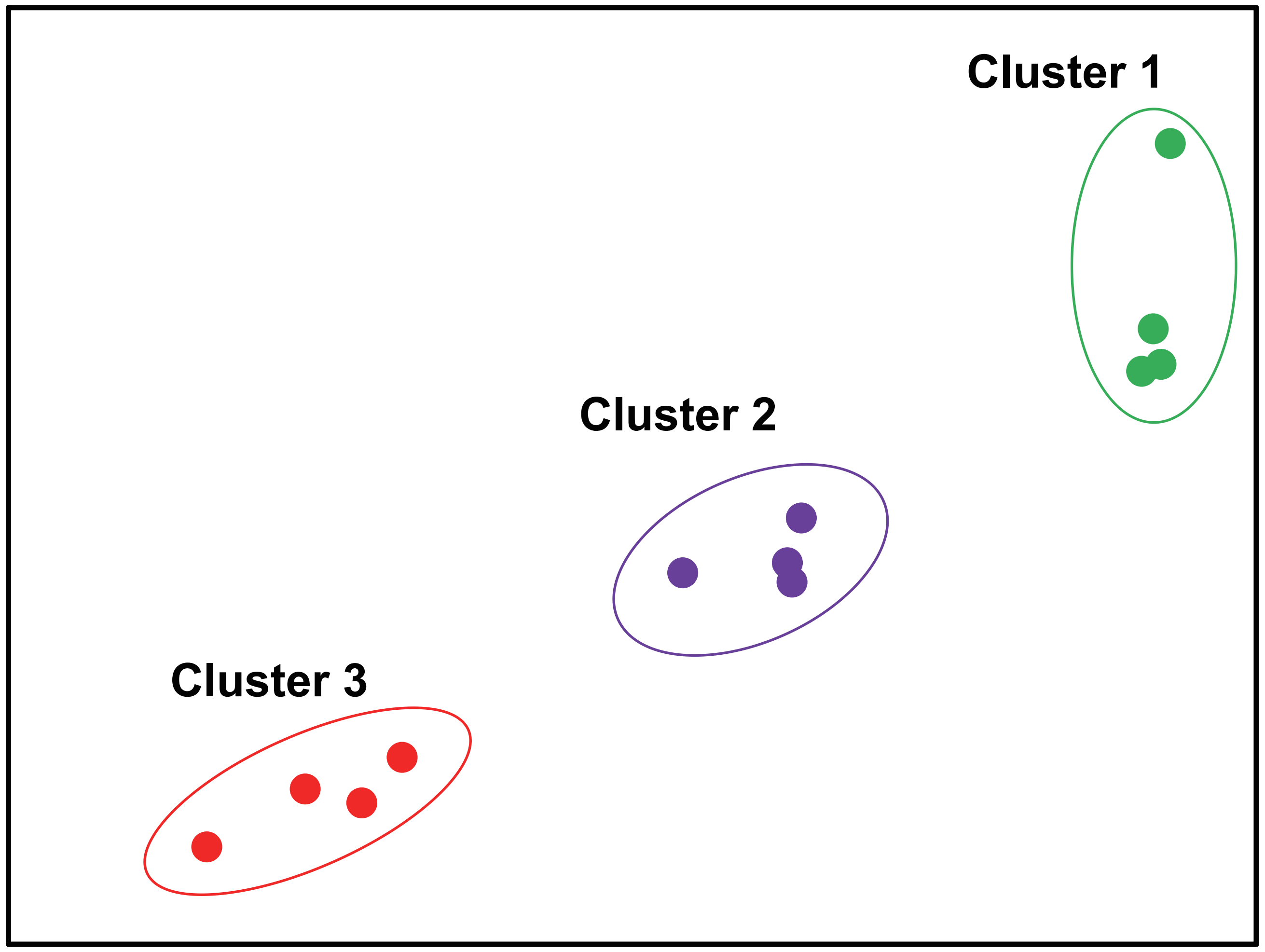

Supplement: Supplementary file 1 [file Data_Sheet_1.zip › Supplementary files/Figure S1.pdf]
