# Supplementary material for: Impact of the soil layer on the soil microbial diversity and composition of Pinus yunnanensis at the Ailao Mountains subtropical forest
Source: Front Microbiol. 2025 May 29;16:1558906. doi: 10.3389/fmicb.2025.1558906 (PMC12159057; doi:10.3389/fmicb.2025.1558906)
Supplement: Supplementary file 1 [file Data_Sheet_1.zip › Supplementary files/Figure S10.pdf]

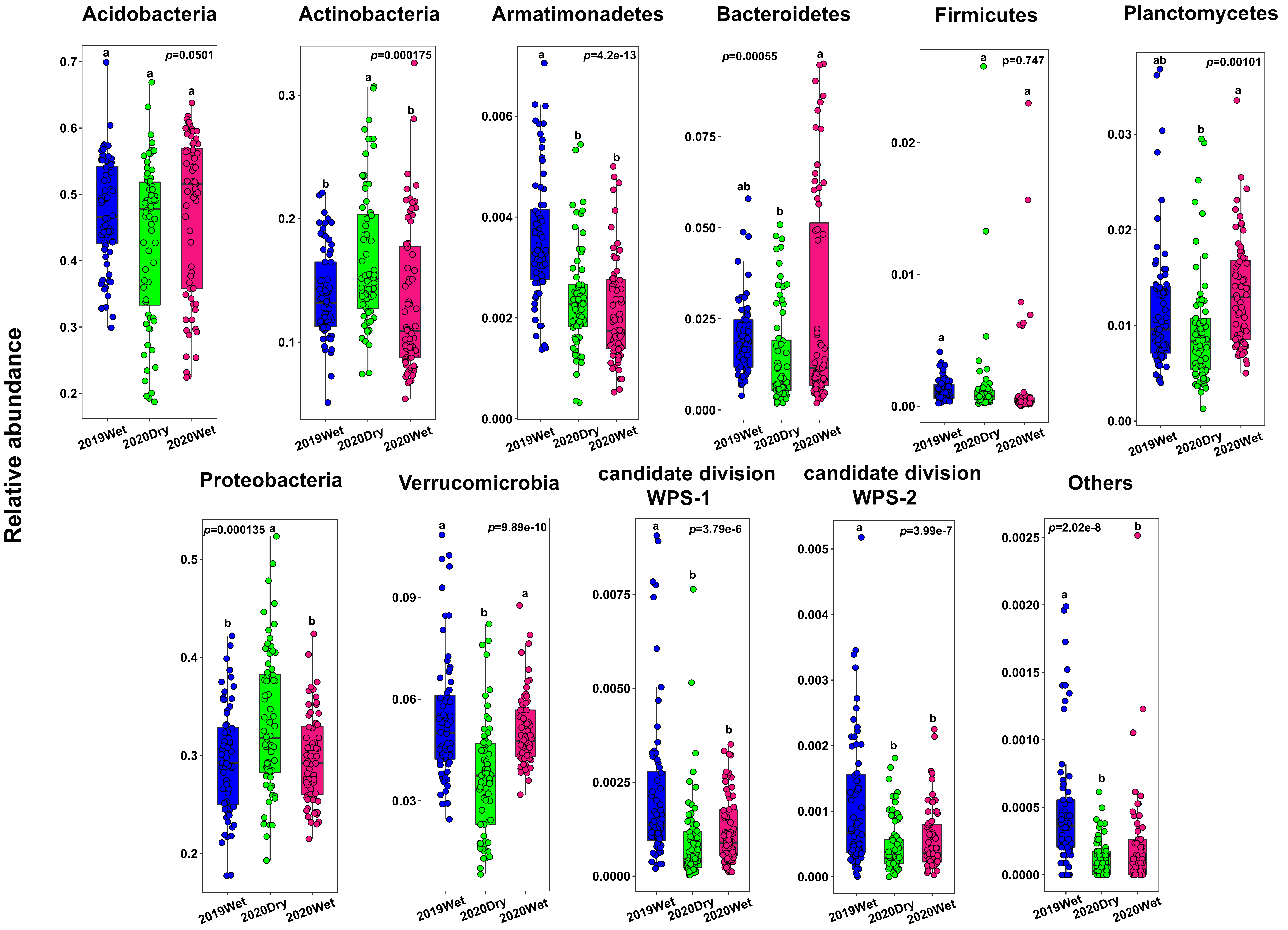

Relative abundance
Acidobacteria
p=0.0501
Actinobacteria
p=0.000175
Armatimonadetes
p=4.2e-13
Bacteroidetes
p=0.00055
Firmicutes
p=0.747
Planctomycetes
p=0.00101
Proteobacteria
p=0.000135
Verrucomicrobia
p=9.89e-10
candidate division WPS-1
p=3.79e-6
candidate division WPS-2
p=3.99e-7
Others
p=2.02e-8
2019Wet
2020Dry
2020Wet
