# Supplementary material for: Impact of the soil layer on the soil microbial diversity and composition of Pinus yunnanensis at the Ailao Mountains subtropical forest
Source: Front Microbiol. 2025 May 29;16:1558906. doi: 10.3389/fmicb.2025.1558906 (PMC12159057; doi:10.3389/fmicb.2025.1558906)
Supplement: Supplementary file 1 [file Data_Sheet_1.zip › Supplementary files/Figure S11.pdf]

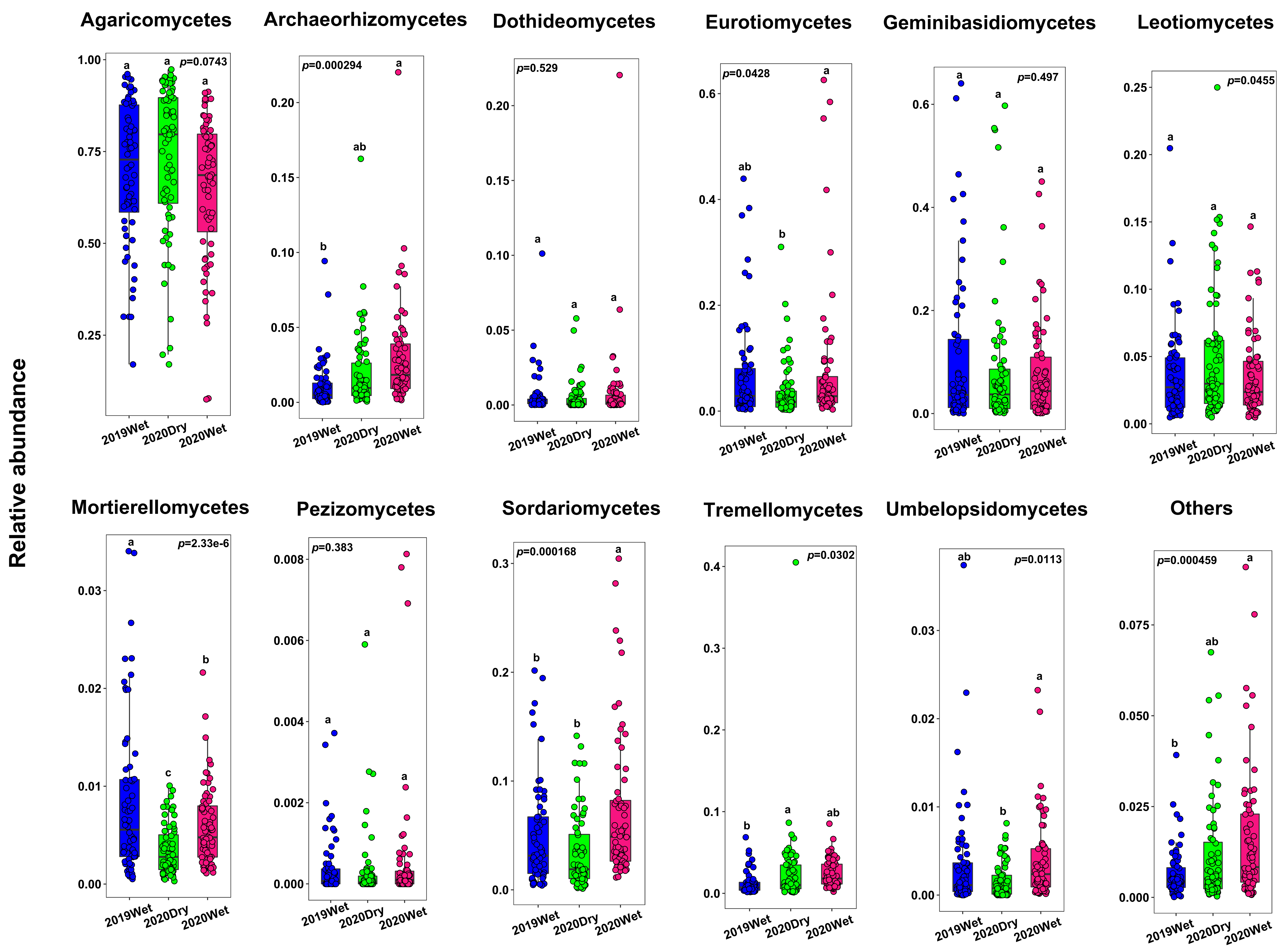

Relative abundance
Agaricomycetes
p=0.0743
Archaeorhizomycetes
p=0.000294
Dothideomycetes
p=0.529
Eurotiomycetes
p=0.0428
Geminibasidiomycetes
p=0.497
Leotiomycetes
p=0.0455
Mortierellomycetes
p=2.33e-6
Pezizomycetes
p=0.383
Sordariomycetes
p=0.000168
Tremellomycetes
p=0.0302
Umbelopsidomycetes
p=0.0113
Others
p=0.000459
2019Wet
2020Dry
2020Wet
