# Supplementary material for: Impact of the soil layer on the soil microbial diversity and composition of Pinus yunnanensis at the Ailao Mountains subtropical forest
Source: Front Microbiol. 2025 May 29;16:1558906. doi: 10.3389/fmicb.2025.1558906 (PMC12159057; doi:10.3389/fmicb.2025.1558906)
Supplement: Supplementary file 1 [file Data_Sheet_1.zip › Supplementary files/Figure S5.pdf]

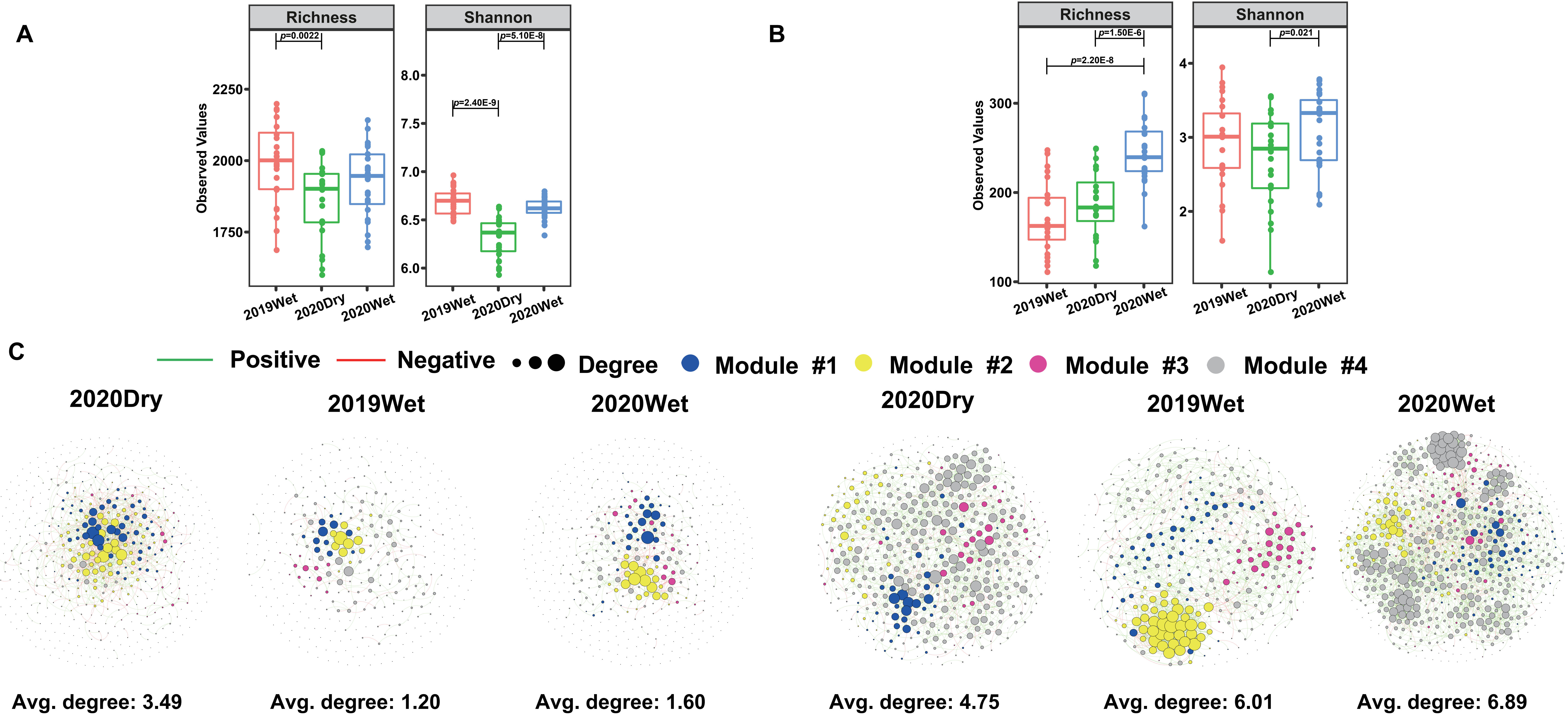
A
Richness
Shannon
p=0.0022
p=5.10E-8
p=2.40E-9
Observed Values
2019Wet
2020Dry
2020Wet
B
Richness
Shannon
p=1.50E-6
p=2.20E-8
p=0.021
Observed Values
C
Positive
Negative
Degree
Module #1
Module #2
Module #3
Module #4
2020Dry
2019Wet
2020Wet
Avg. degree: 3.49
Avg. degree: 1.20
Avg. degree: 1.60
Avg. degree: 4.75
Avg. degree: 6.01
Avg. degree: 6.89
