# Supplementary material for: Impact of the soil layer on the soil microbial diversity and composition of Pinus yunnanensis at the Ailao Mountains subtropical forest
Source: Front Microbiol. 2025 May 29;16:1558906. doi: 10.3389/fmicb.2025.1558906 (PMC12159057; doi:10.3389/fmicb.2025.1558906)
Supplement: Supplementary file 1 [file Data_Sheet_1.zip › Supplementary files/Figure S6.pdf]

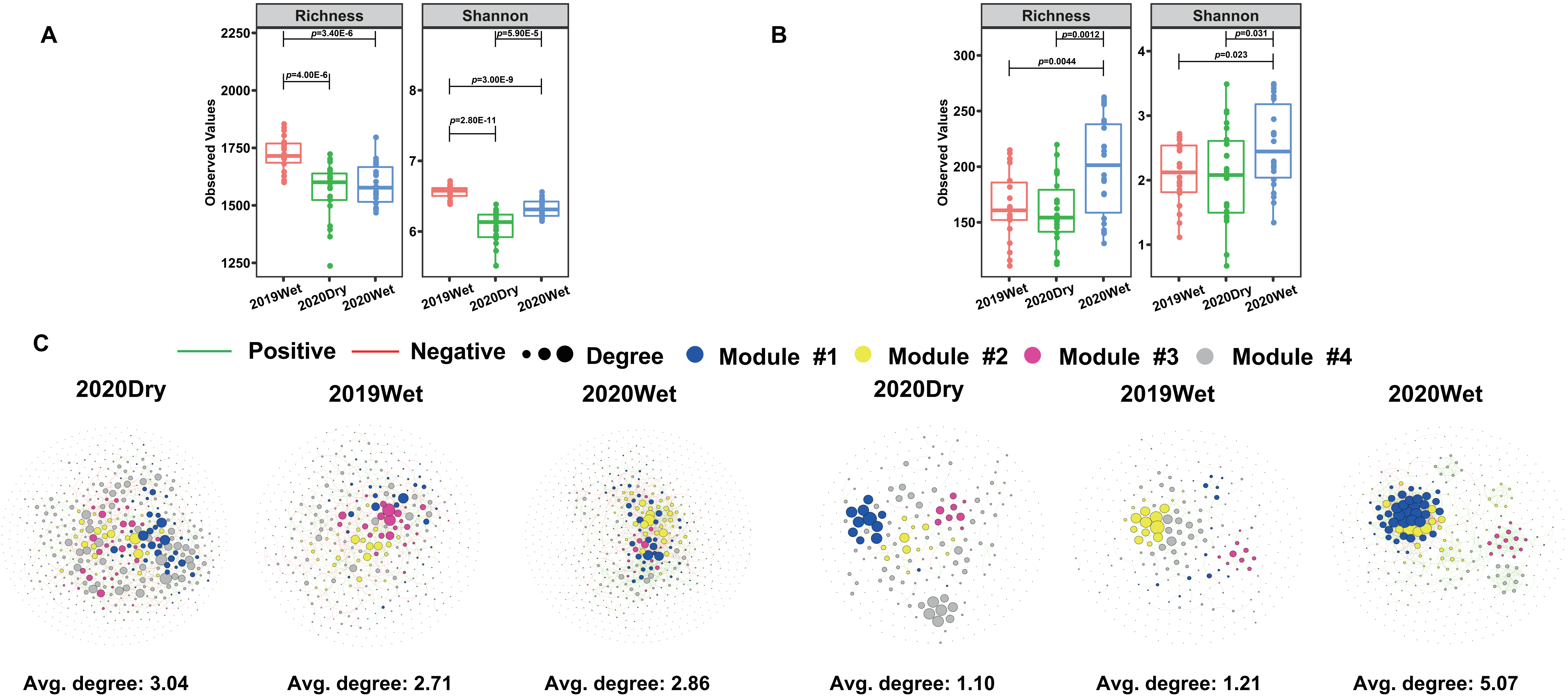
A
Richness
Shannon
p=3.40E-6
p=4.00E-6
p=5.90E-5
p=3.00E-9
p=2.80E-11
Observed Values
2250
2000
1750
1500
1250
8
7
6
2019Wet
2020Dry
2020Wet
B
Richness
Shannon
p=0.0012
p=0.0044
p=0.031
p=0.023
300
250
200
150
4
3
2
1
C
Positive
Negative
Degree
Module #1
Module #2
Module #3
Module #4
2020Dry
2019Wet
2020Wet
Avg. degree: 3.04
Avg. degree: 2.71
Avg. degree: 2.86
Avg. degree: 1.10
Avg. degree: 1.21
Avg. degree: 5.07
