# Supplementary material for: Impact of the soil layer on the soil microbial diversity and composition of Pinus yunnanensis at the Ailao Mountains subtropical forest
Source: Front Microbiol. 2025 May 29;16:1558906. doi: 10.3389/fmicb.2025.1558906 (PMC12159057; doi:10.3389/fmicb.2025.1558906)
Supplement: Supplementary file 1 [file Data_Sheet_1.zip › Supplementary files/Figure S7.pdf]

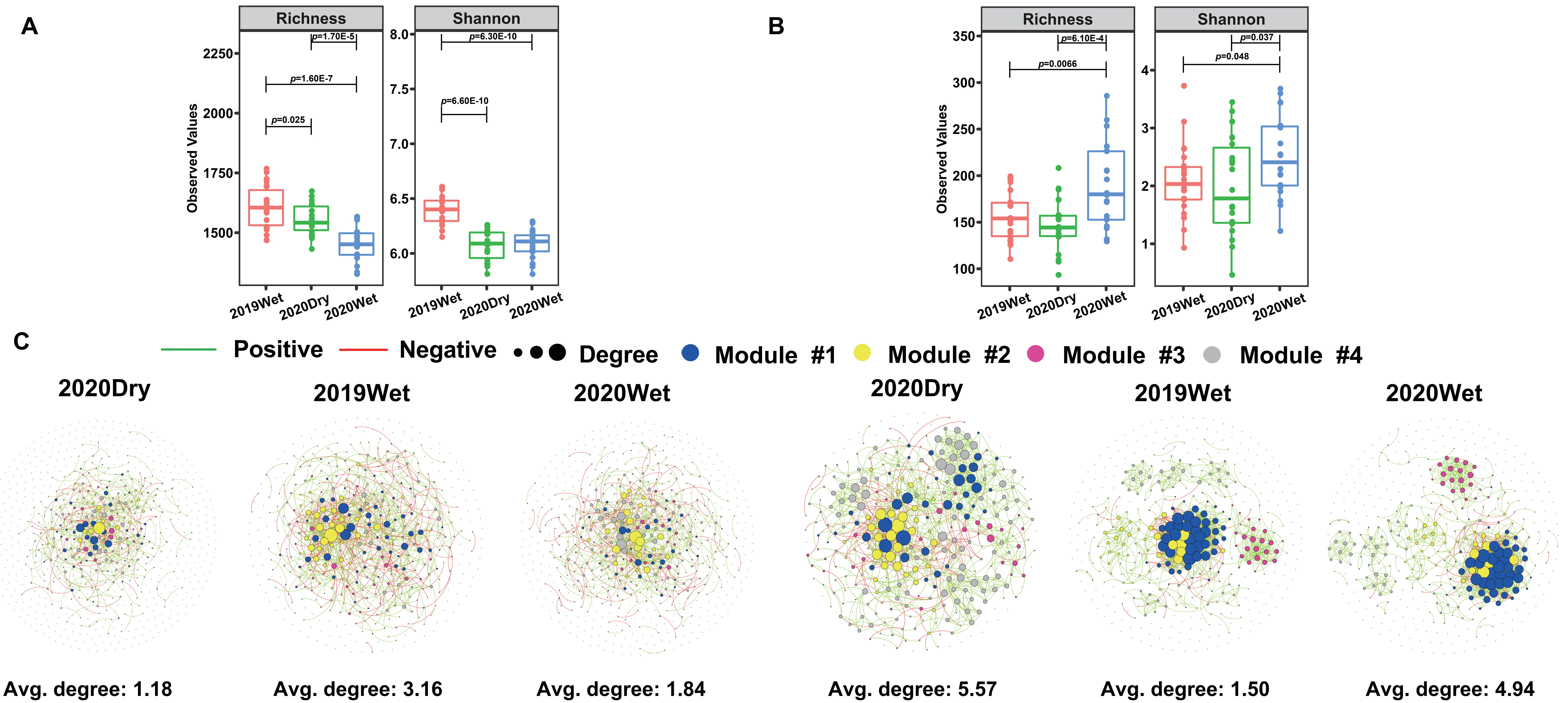
A
Richness
Shannon
Observed Values
p=1.70E-5
p=1.60E-7
p=0.025
p=6.30E-10
p=6.60E-10
2019Wet
2020Dry
2020Wet
B
Richness
Shannon
p=6.10E-4
p=0.0066
p=0.037
p=0.048
C
Positive
Negative
Degree
Module #1
Module #2
Module #3
Module #4
2020Dry
2019Wet
2020Wet
Avg. degree: 1.18
Avg. degree: 3.16
Avg. degree: 1.84
Avg. degree: 5.57
Avg. degree: 1.50
Avg. degree: 4.94
