# Supplementary material for: Impact of the soil layer on the soil microbial diversity and composition of Pinus yunnanensis at the Ailao Mountains subtropical forest
Source: Front Microbiol. 2025 May 29;16:1558906. doi: 10.3389/fmicb.2025.1558906 (PMC12159057; doi:10.3389/fmicb.2025.1558906)
Supplement: Supplementary file 1 [file Data_Sheet_1.zip › Supplementary files/Figure S8.pdf]

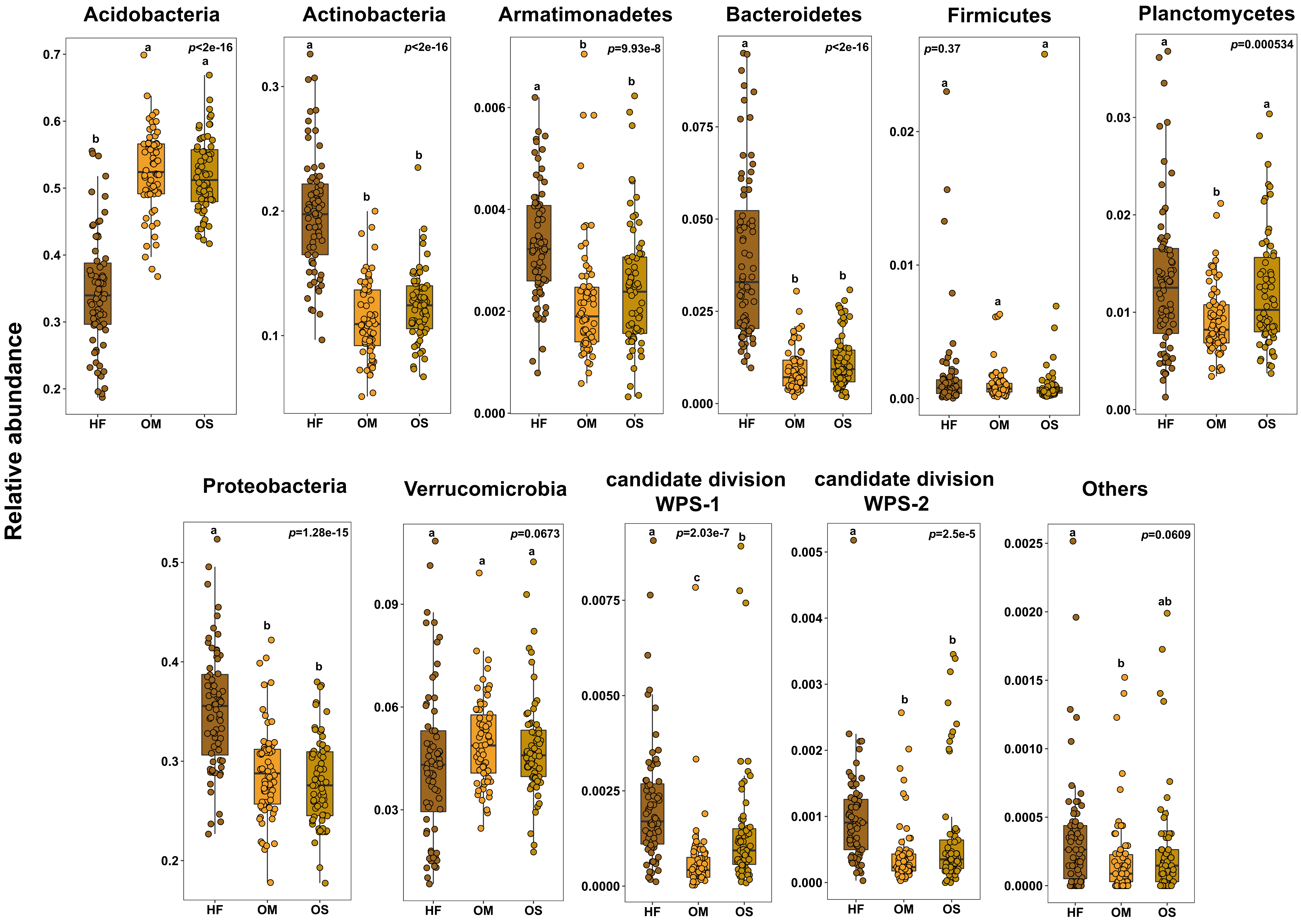
Acidobacteria
p<2e-16
Actinobacteria
p<2e-16
Armatimonadetes
p=9.93e-8
Bacteroidetes
p<2e-16
Firmicutes
p=0.37
Planctomycetes
p=0.000534
Proteobacteria
p=1.28e-15
Verrucomicrobia
p=0.0673
candidate division WPS-1
p=2.03e-7
candidate division WPS-2
p=2.5e-5
Others
p=0.0609
Relative abundance
HF
OM
OS
