# Supplementary material for: Impact of the soil layer on the soil microbial diversity and composition of Pinus yunnanensis at the Ailao Mountains subtropical forest
Source: Front Microbiol. 2025 May 29;16:1558906. doi: 10.3389/fmicb.2025.1558906 (PMC12159057; doi:10.3389/fmicb.2025.1558906)
Supplement: Supplementary file 1 [file Data_Sheet_1.zip › Supplementary files/Figure S9.pdf]

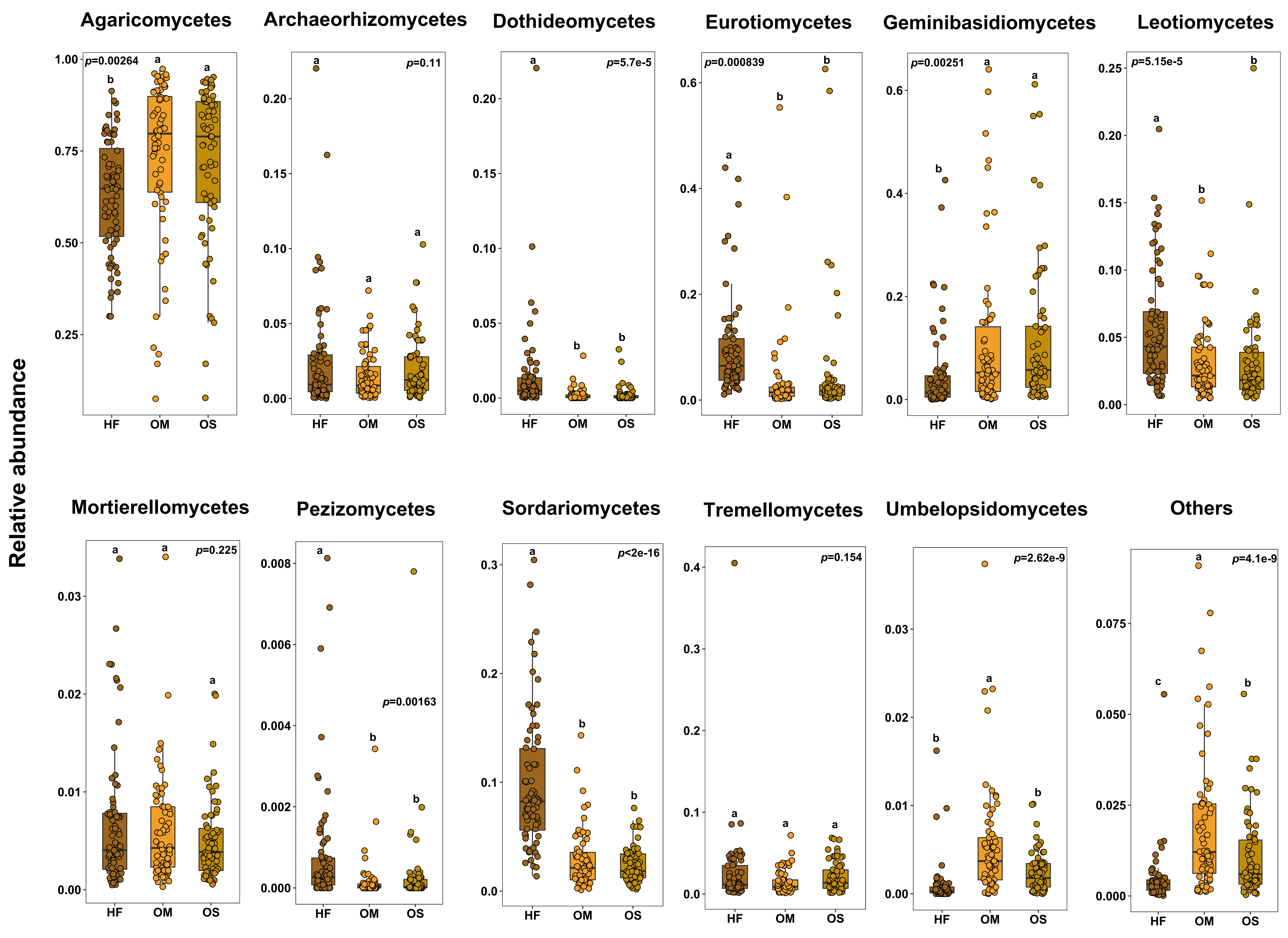

Relative abundance
Agaricomycetes
p=0.00264
Archaeorhizomycetes
p=0.11
Dothideomycetes
p=5.7e-5
Eurotiomycetes
p=0.000839
Geminibasidiomycetes
p=0.00251
Leotiomycetes
p=5.15e-5
Mortierellomycetes
p=0.225
Pezizomycetes
p=0.00163
Sordariomycetes
p<2e-16
Tremellomycetes
p=0.154
Umbelopsidomycetes
p=2.62e-9
Others
p=4.1e-9
HF
OM
OS
