# Supplementary figures and images for: Impact of the soil layer on the soil microbial diversity and composition of Pinus yunnanensis at the Ailao Mountains subtropical forest
Source: Front Microbiol. 2025 May 29;16:1558906. doi: 10.3389/fmicb.2025.1558906 (PMC12159057; doi:10.3389/fmicb.2025.1558906)

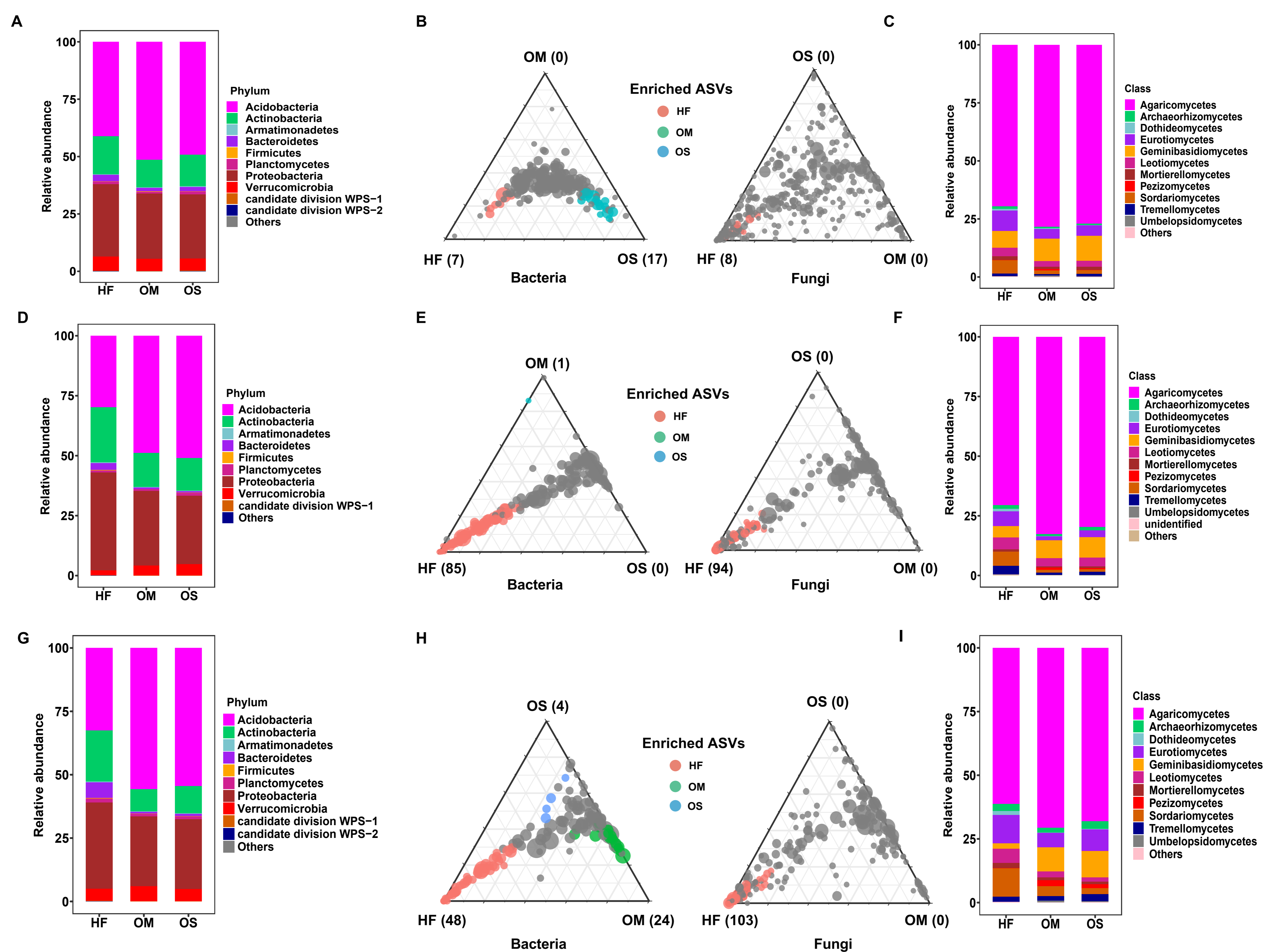

Supplement: Supplementary file 1 [file Data_Sheet_1.zip › Supplementary files/Figure S12.pdf]

A

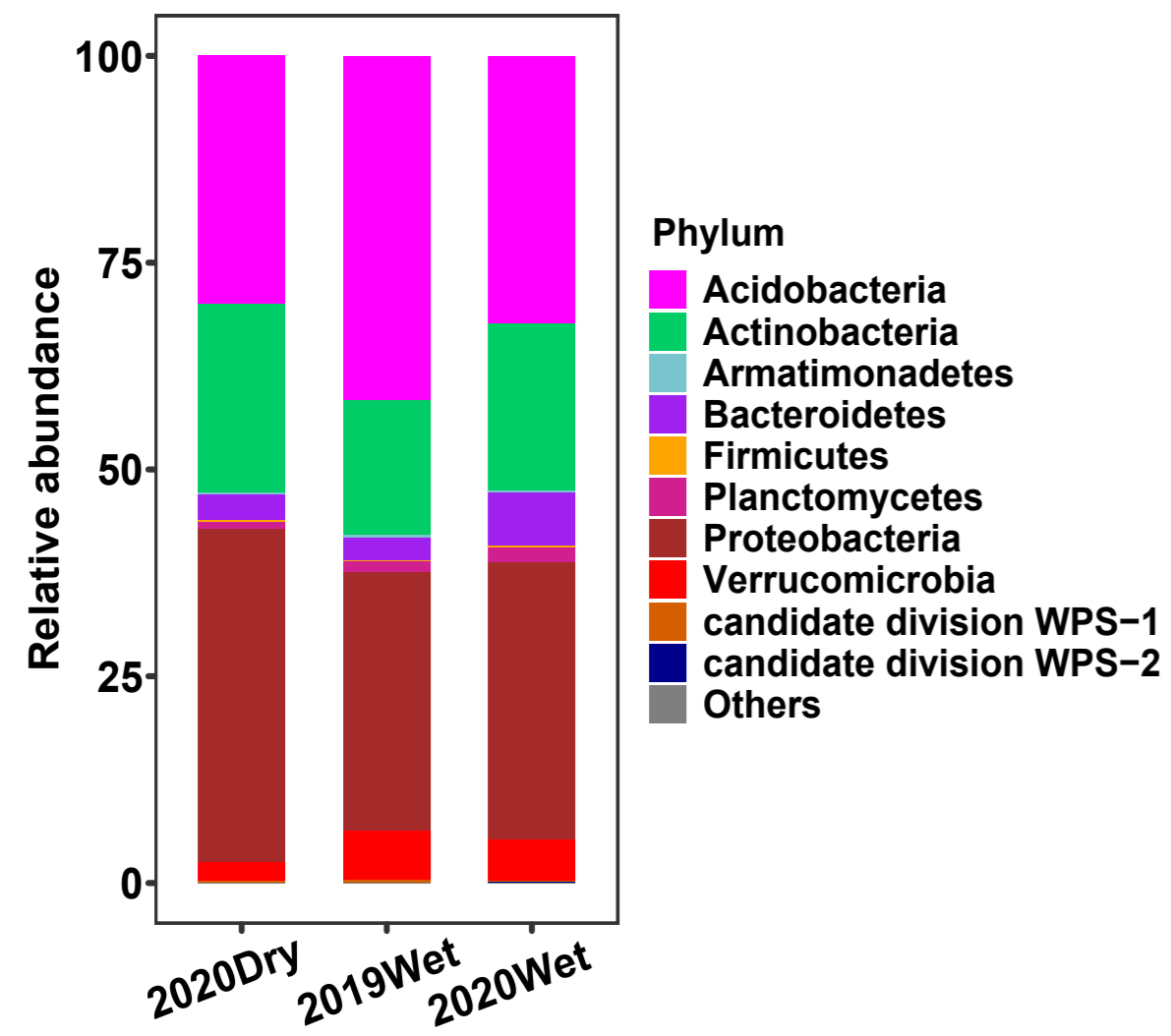

B

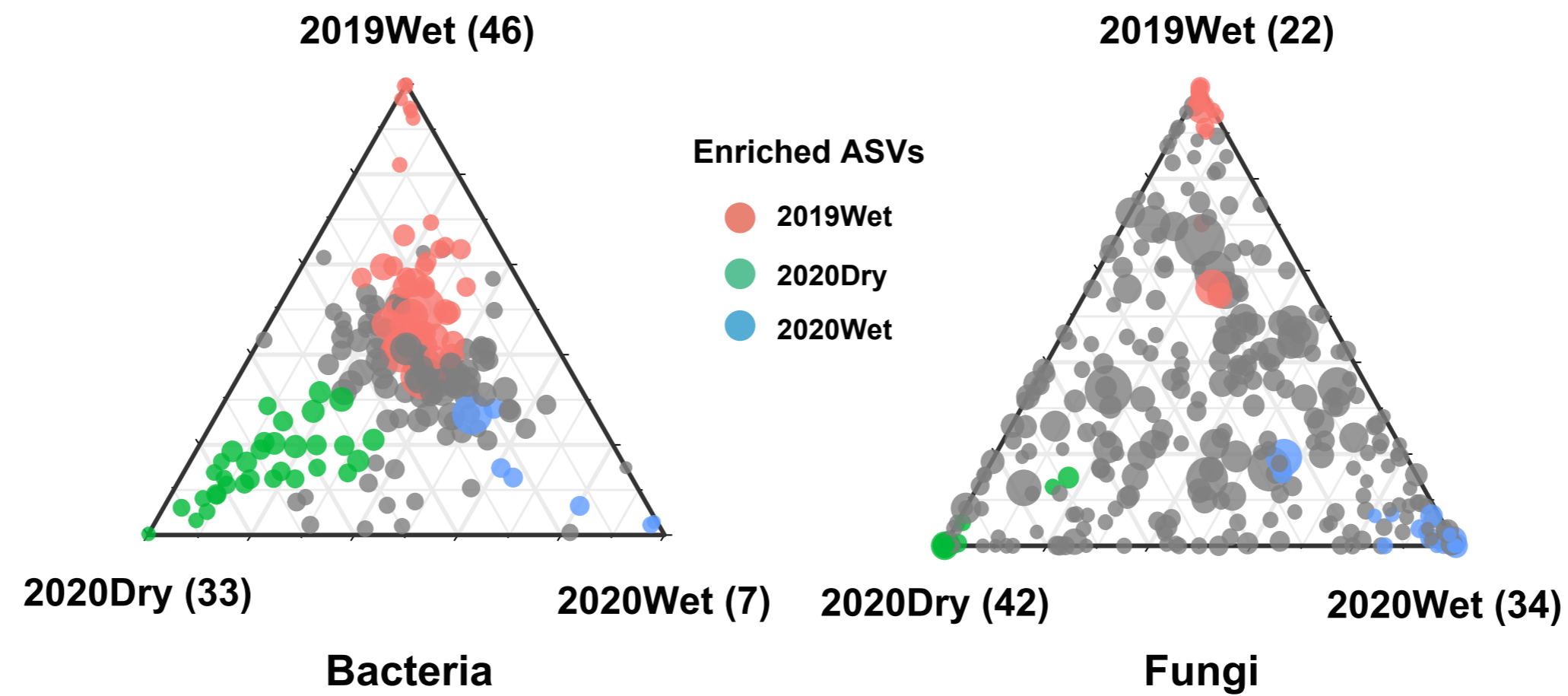

C

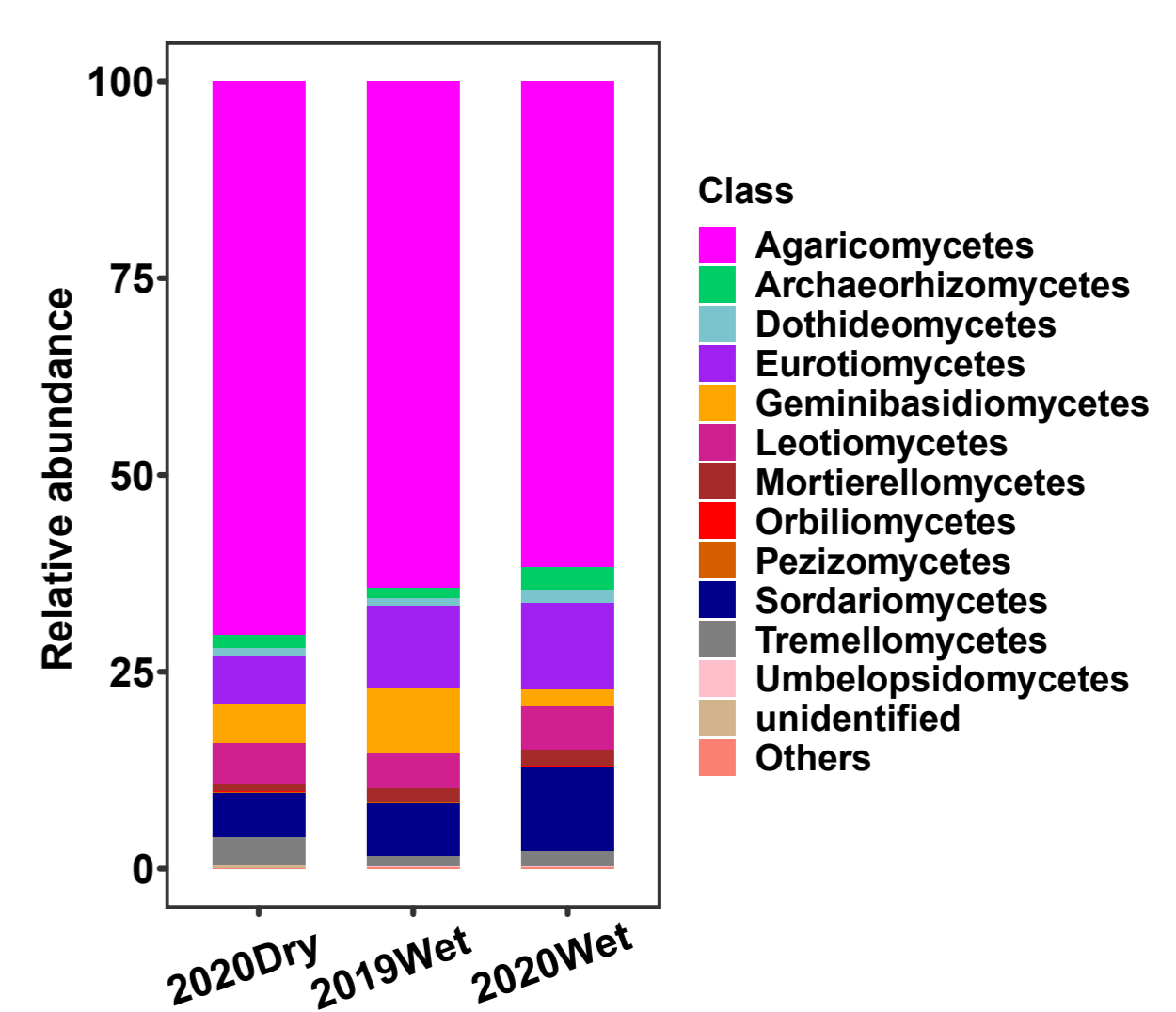

D

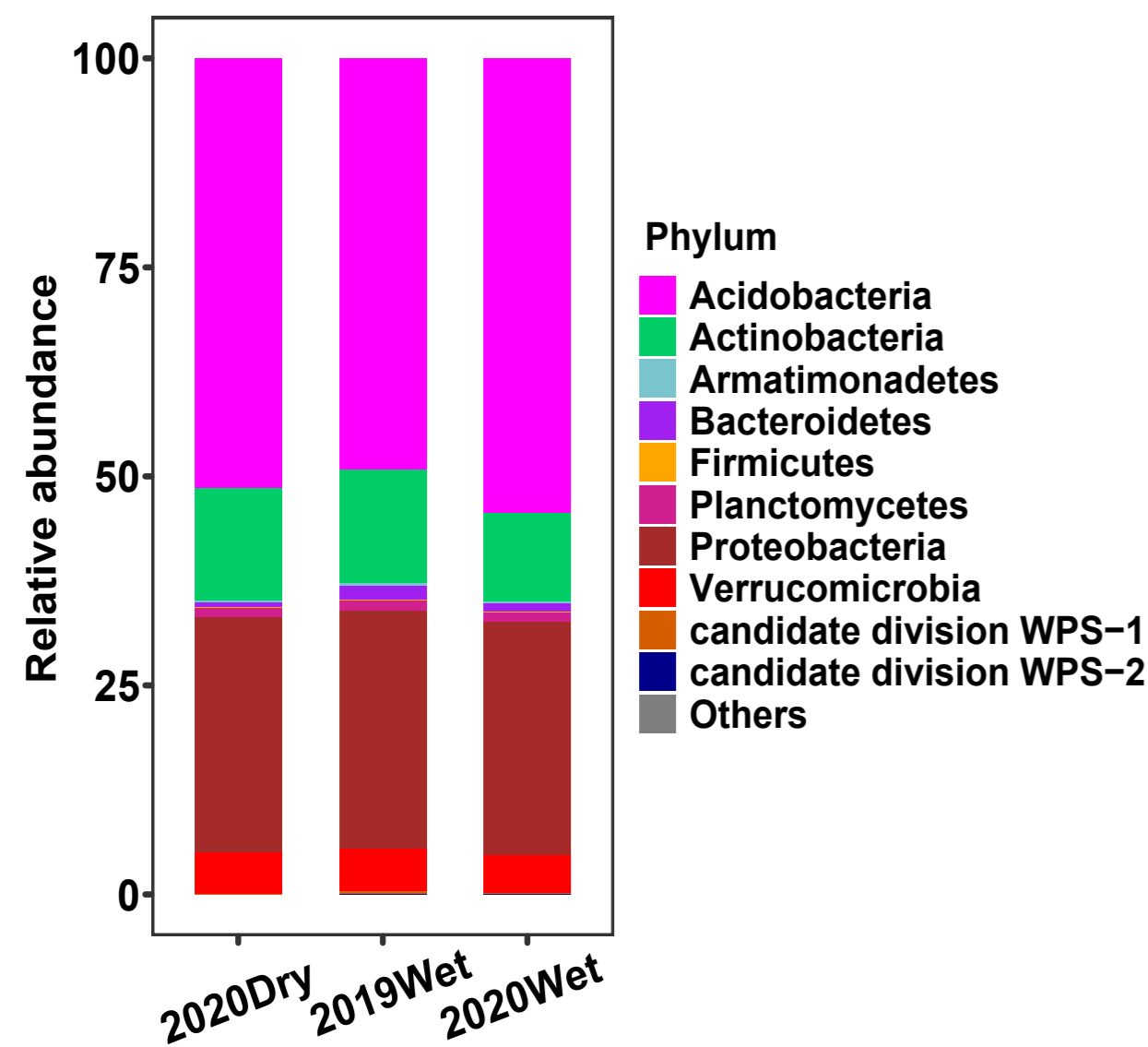

E

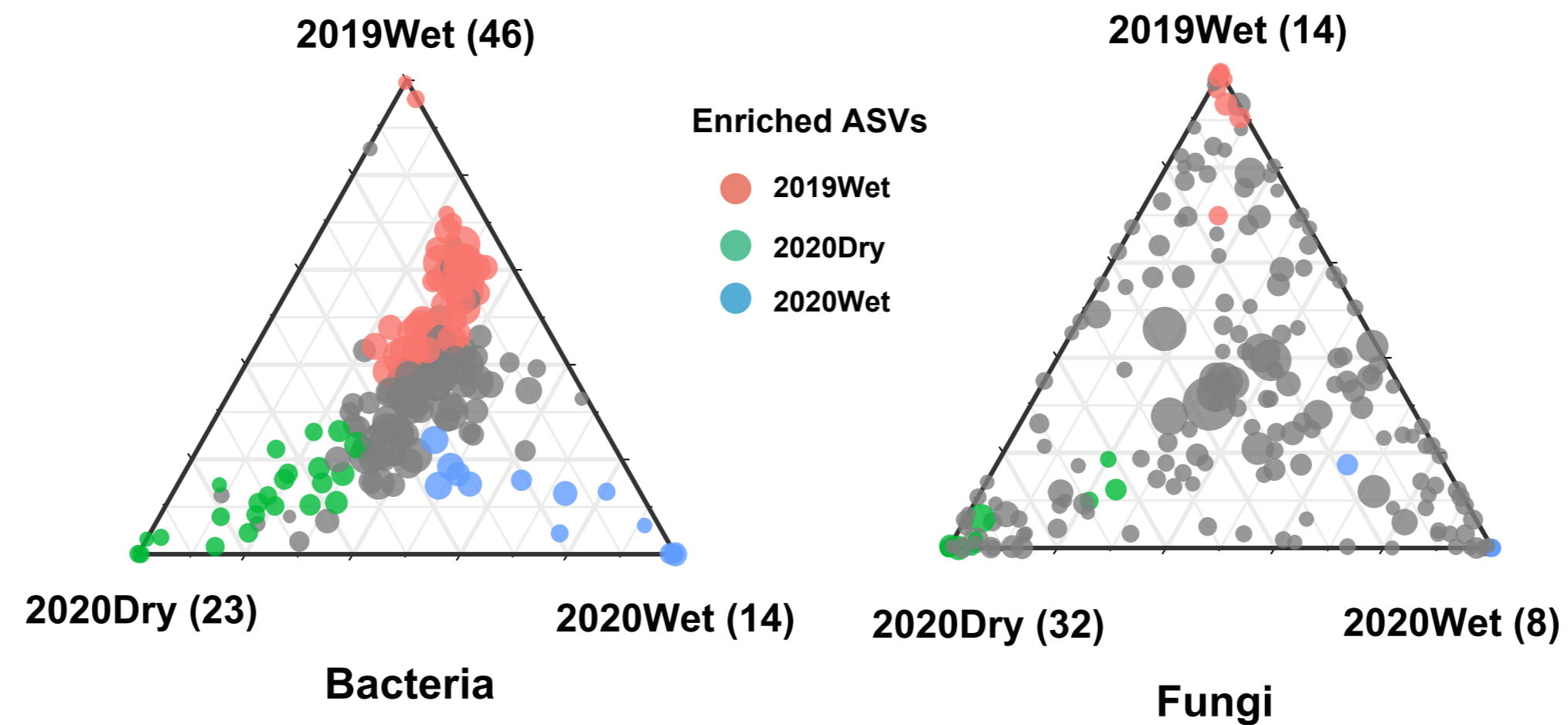

F

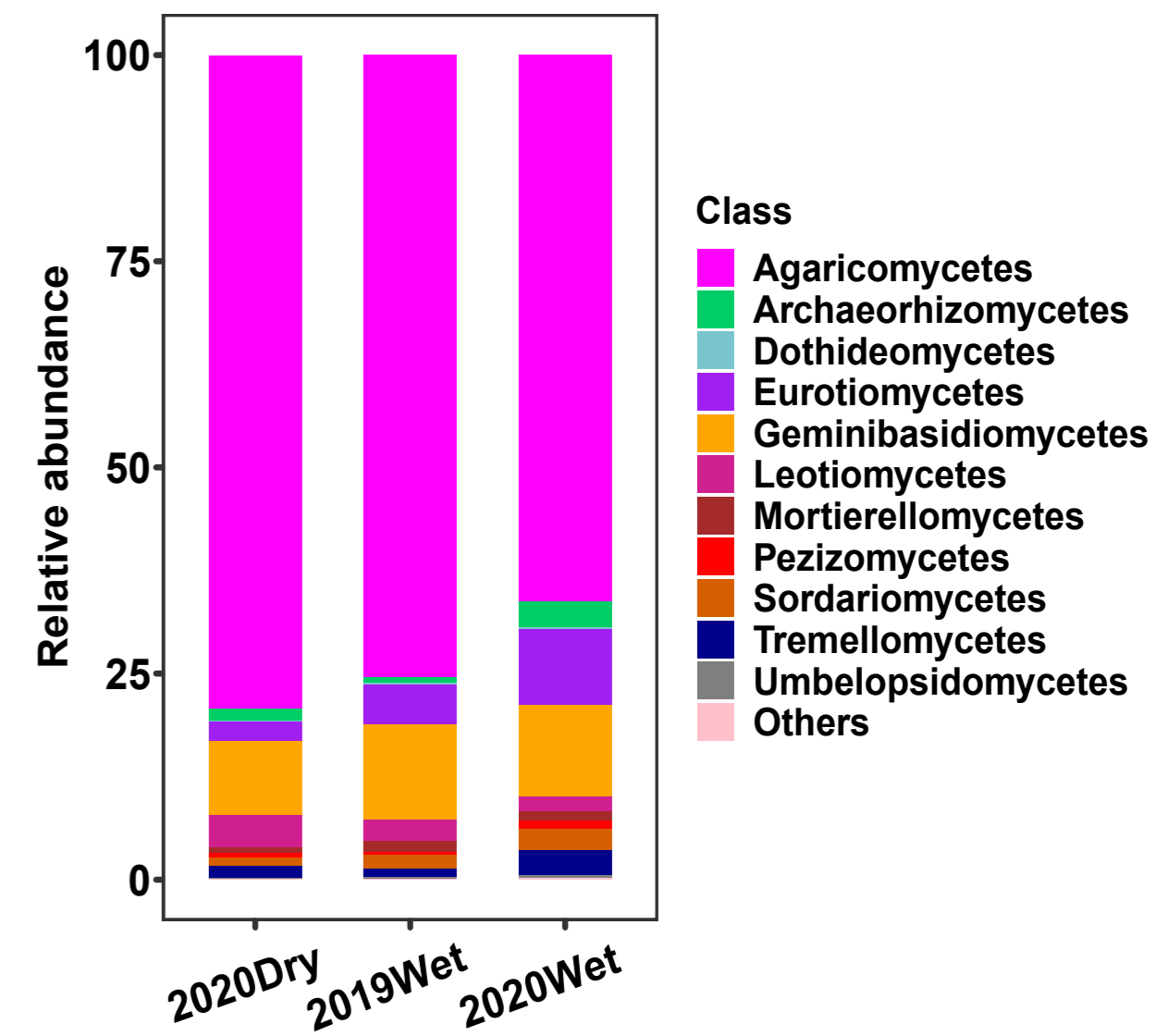

G

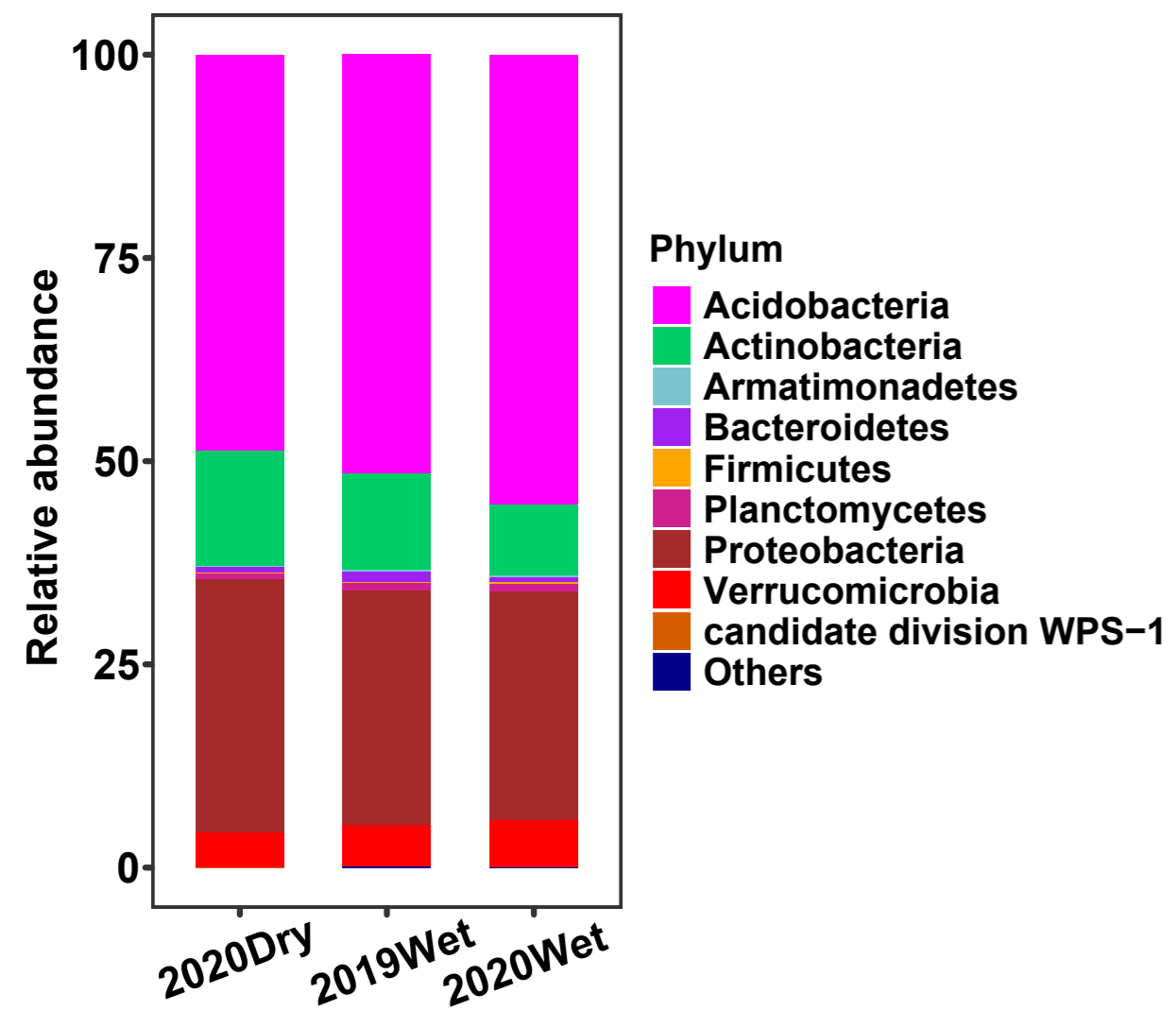

H

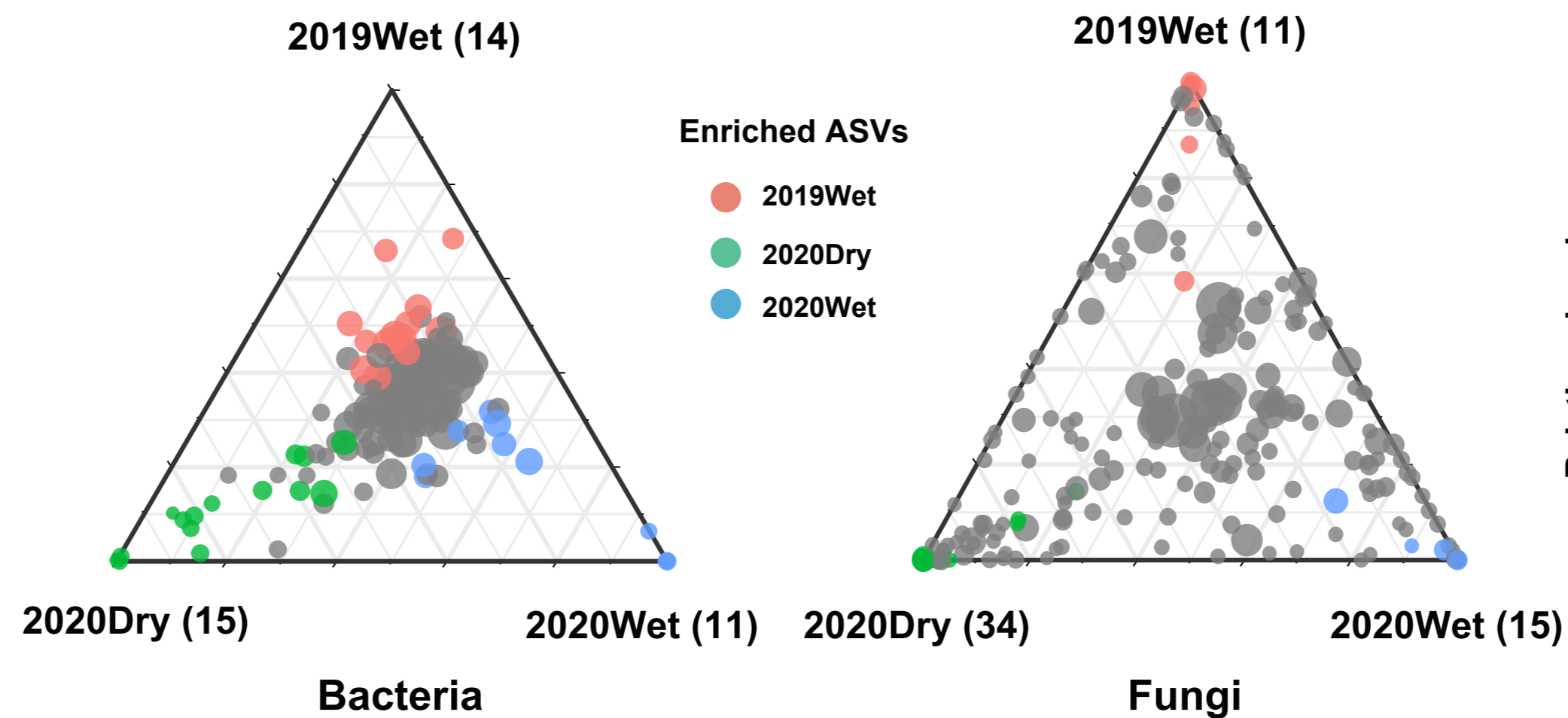

I

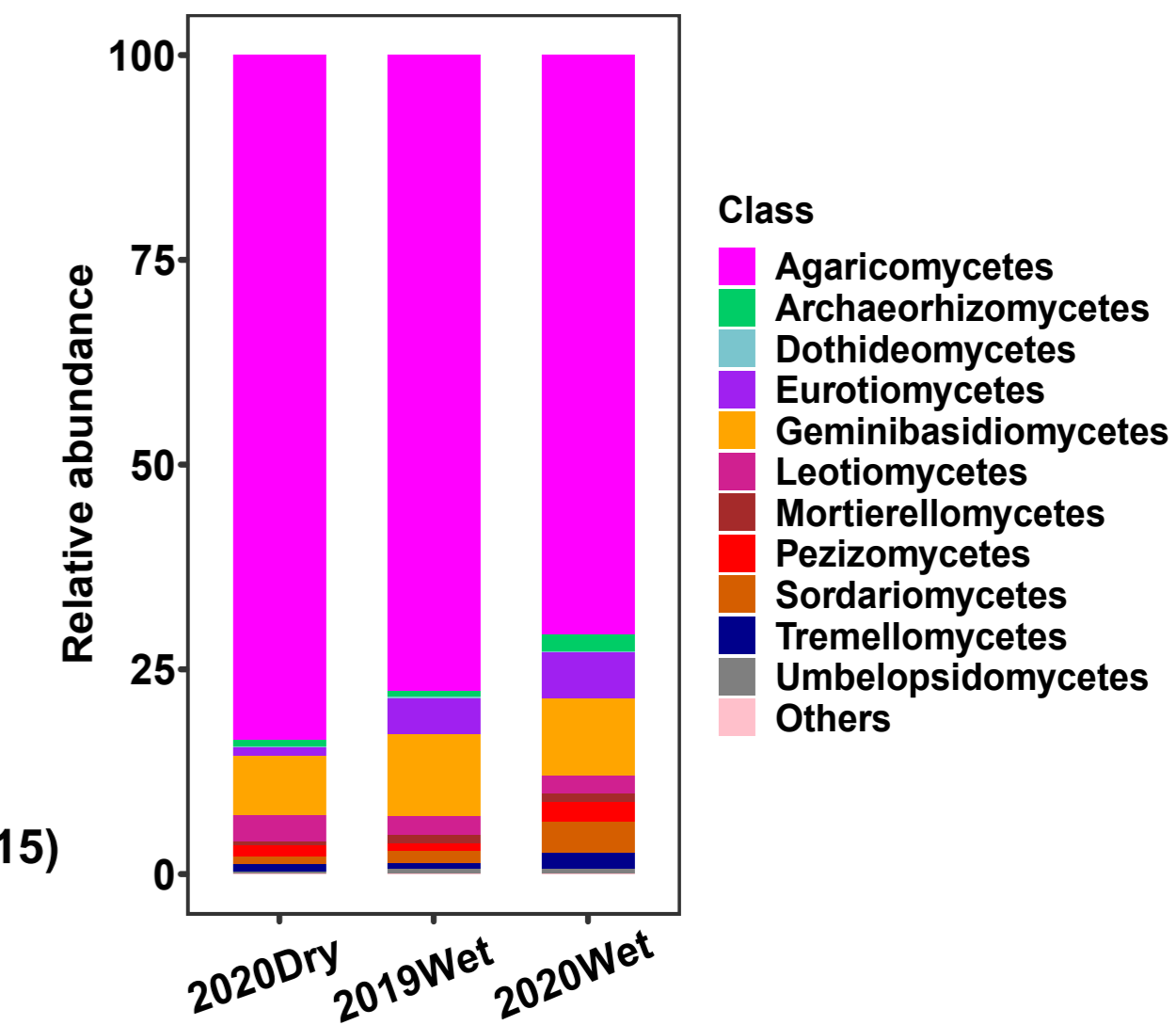

Supplement: Supplementary file 1 [file Data_Sheet_1.zip › Supplementary files/Figure S13.pdf]

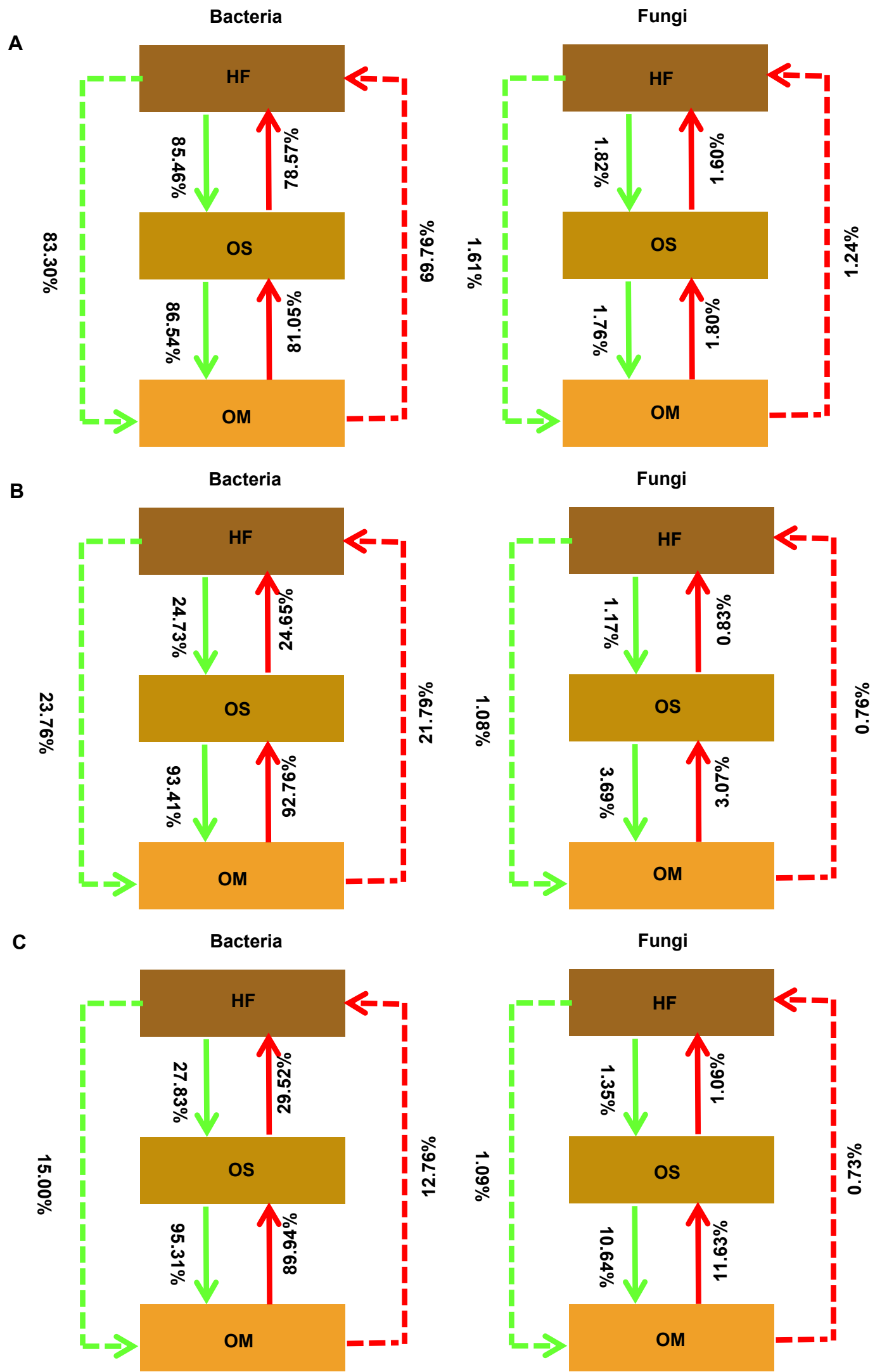

Supplement: Supplementary file 1 [file Data_Sheet_1.zip › Supplementary files/Figure S14.pdf]

A

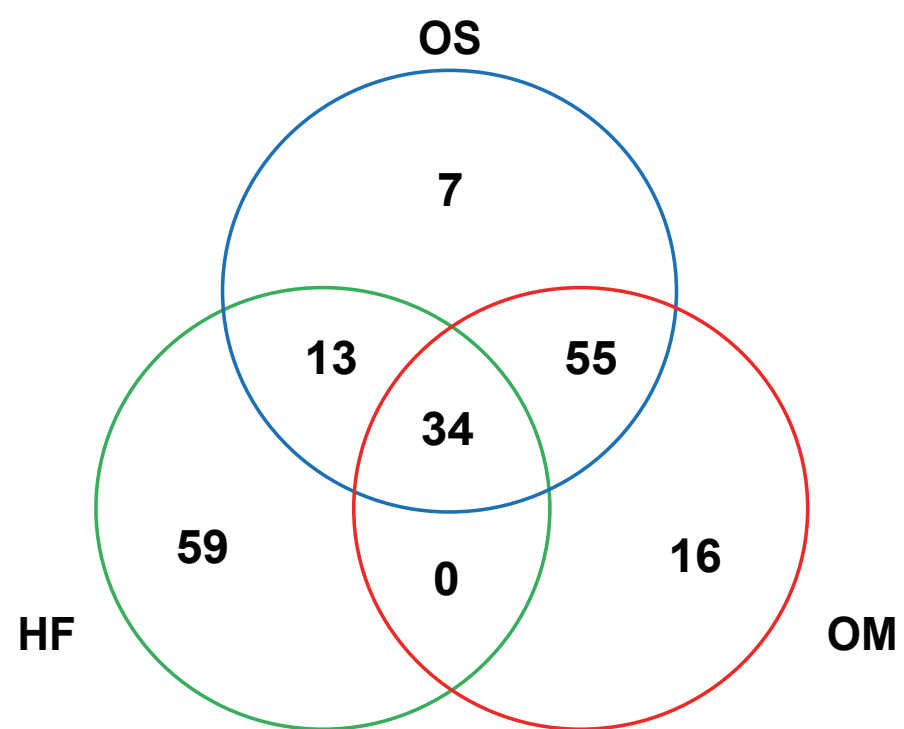

B

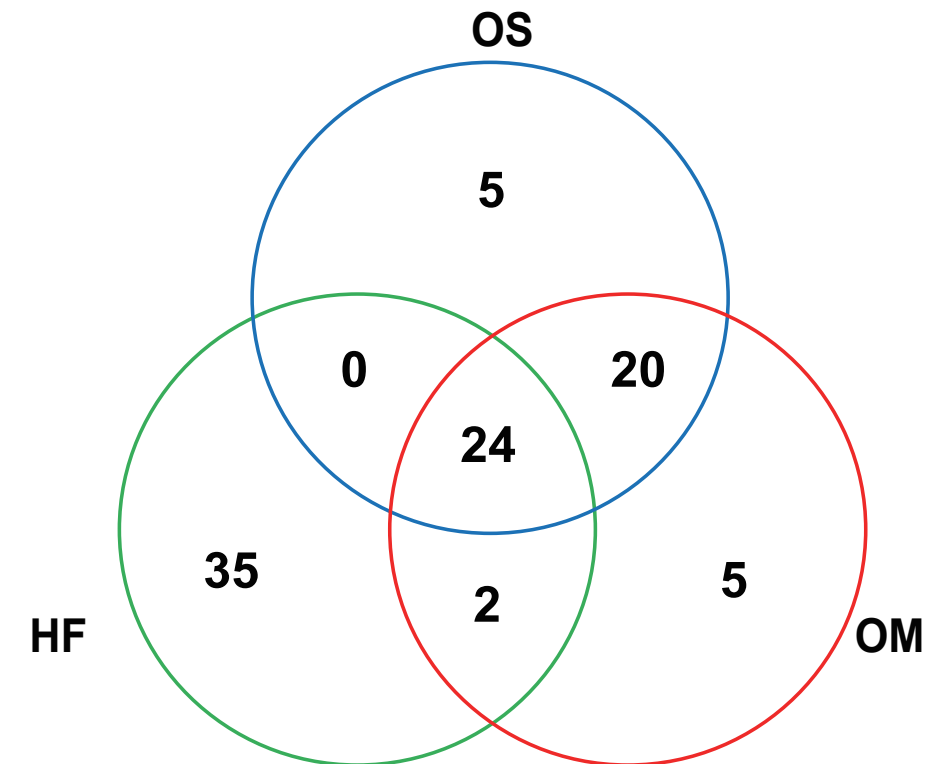

C

■ HF ■ OS ■ OM

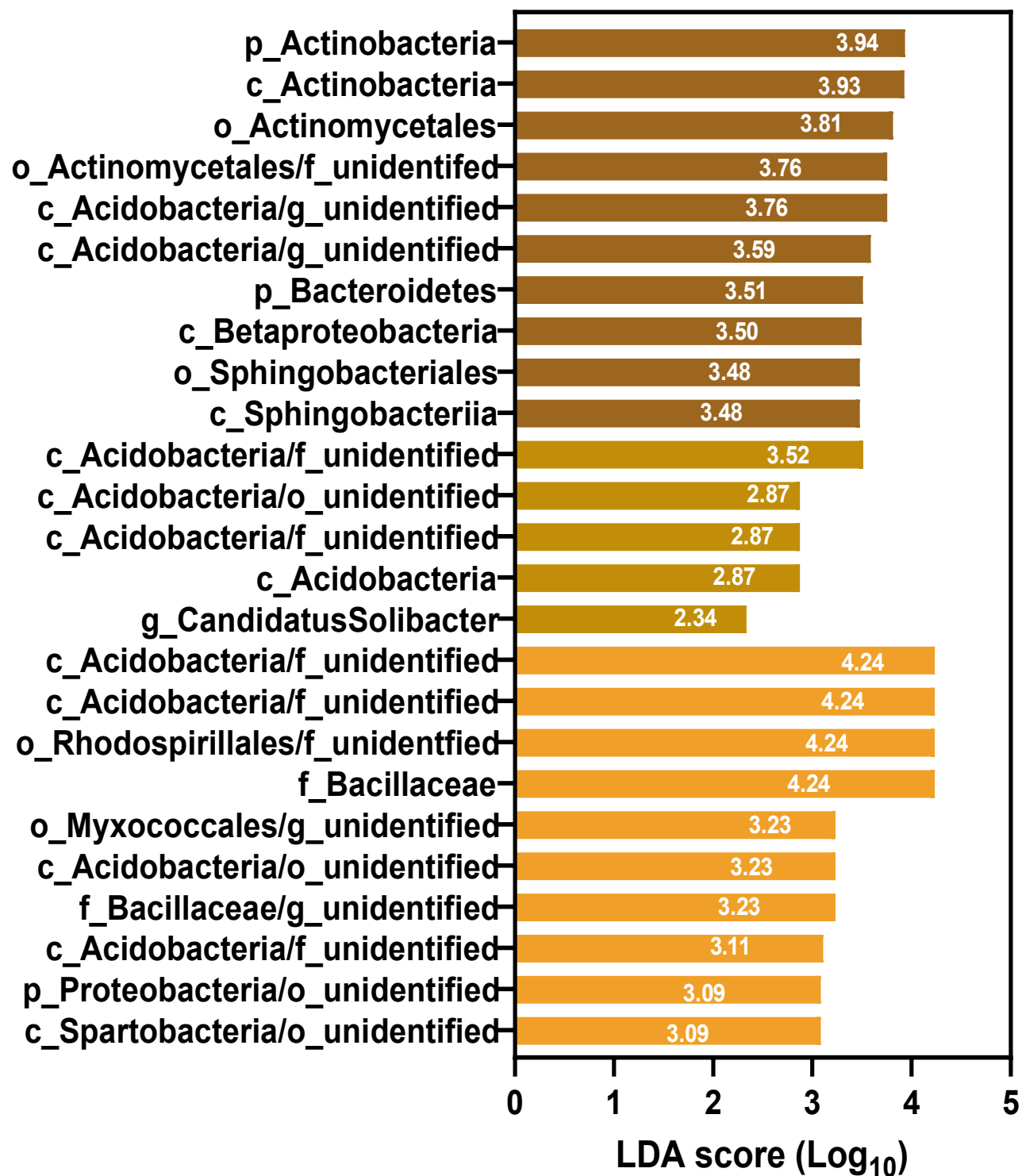

D

■ HF ■ OS ■ OM

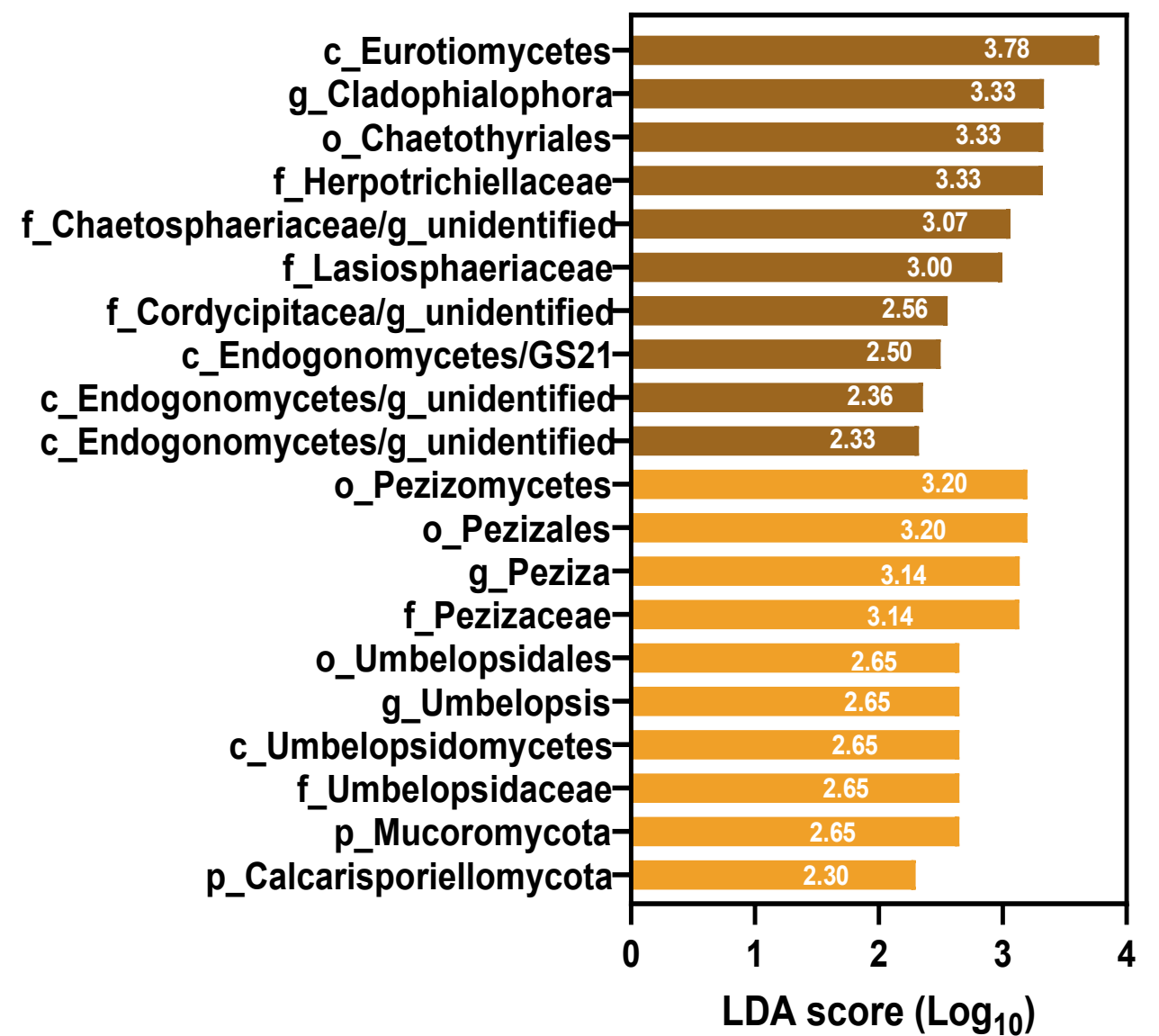

Supplement: Supplementary file 1 [file Data_Sheet_1.zip › Supplementary files/Figure S15.pdf]

A

2020Wet

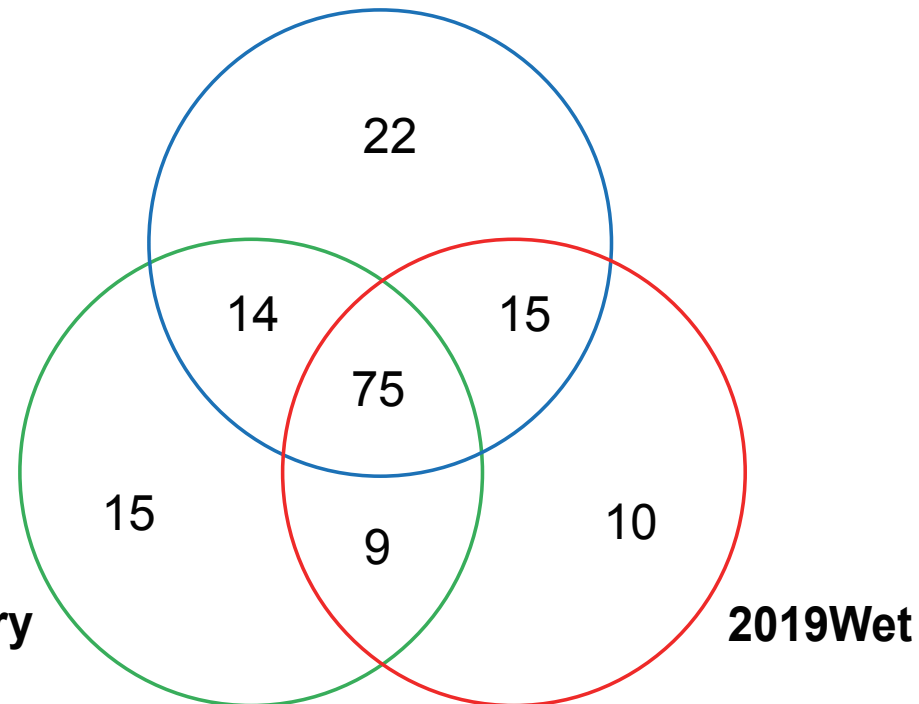

B

2020Wet

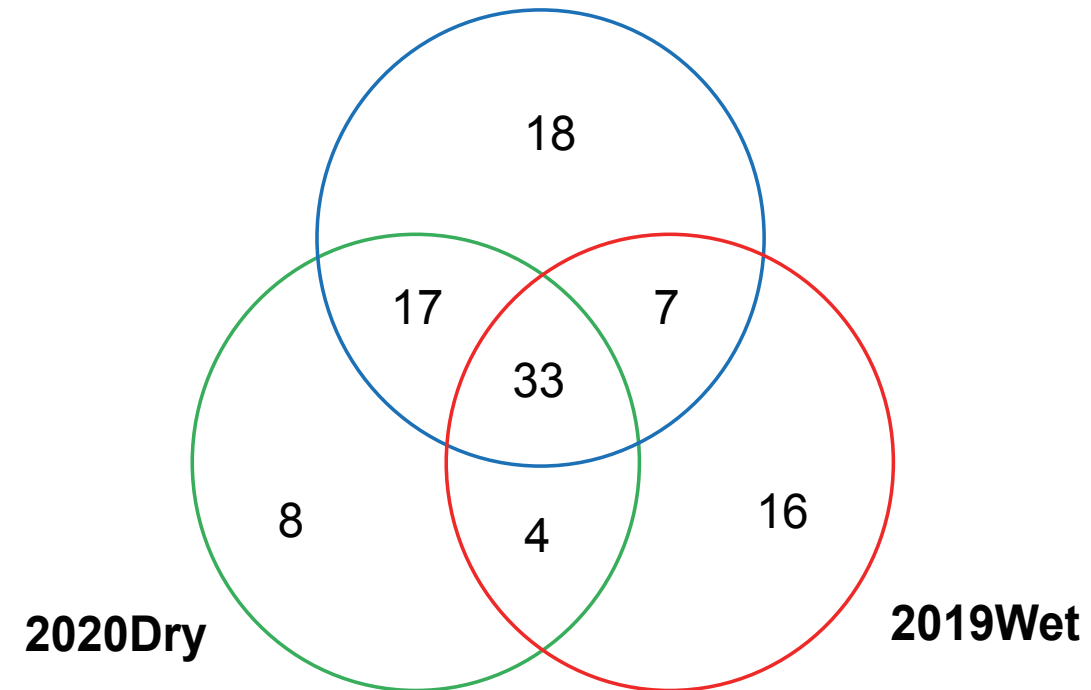

C

2020Dry 2019Wet 2020Wet

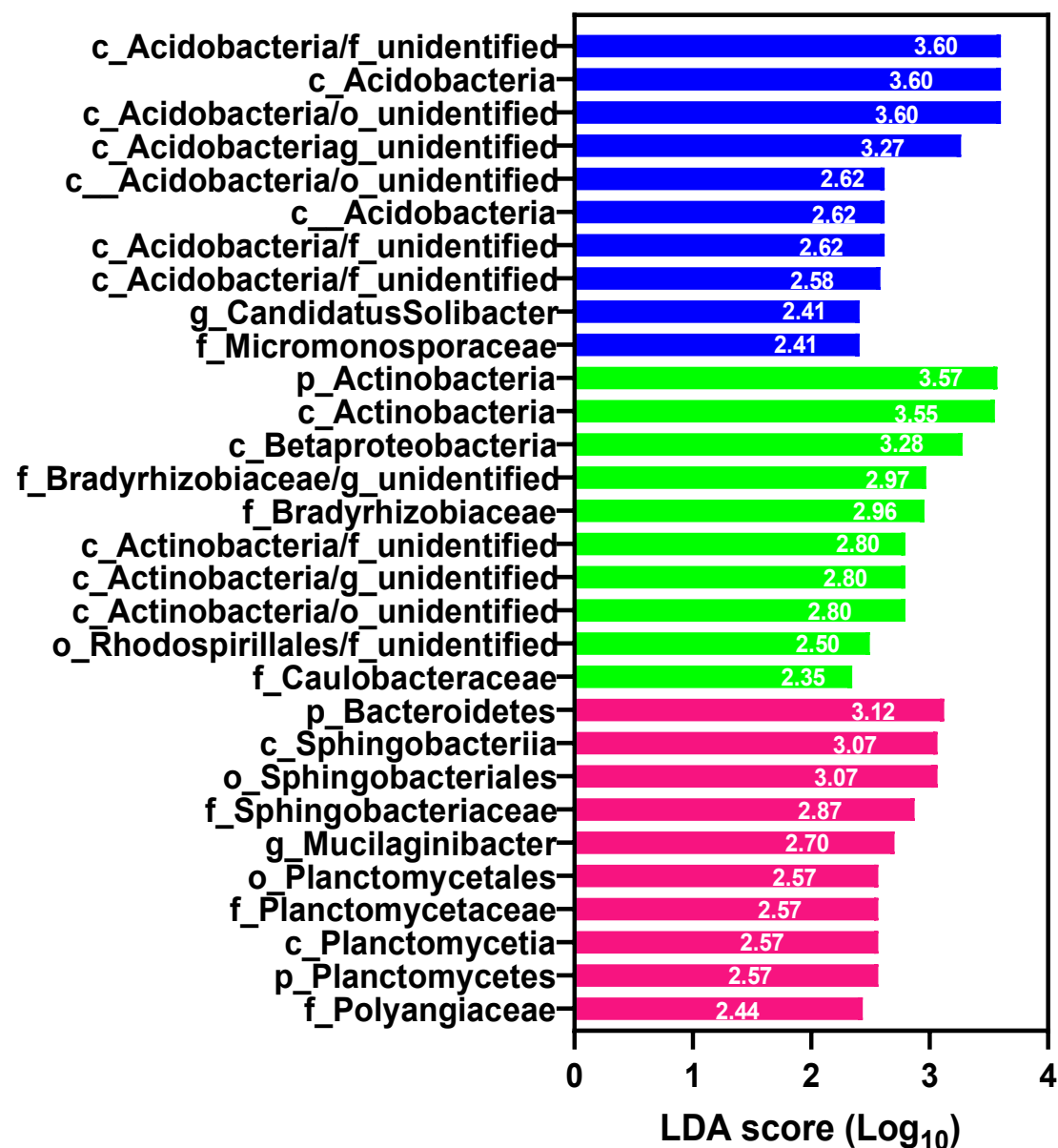

D

2020Dry 2019Wet 2020Wet

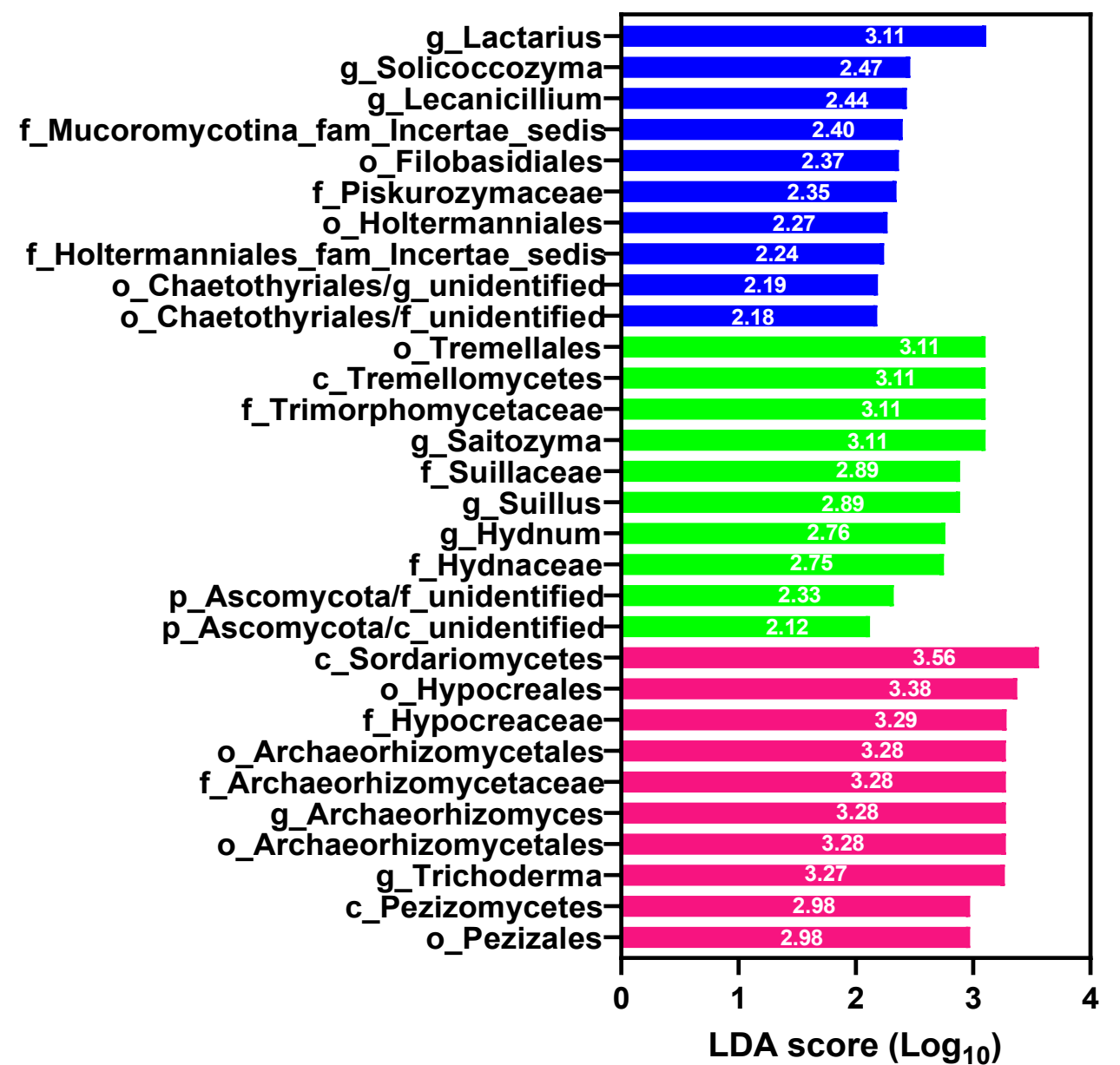

Supplement: Supplementary file 1 [file Data_Sheet_1.zip › Supplementary files/Figure S18.pdf]

## Bacteria

## Fungi

2019Wet

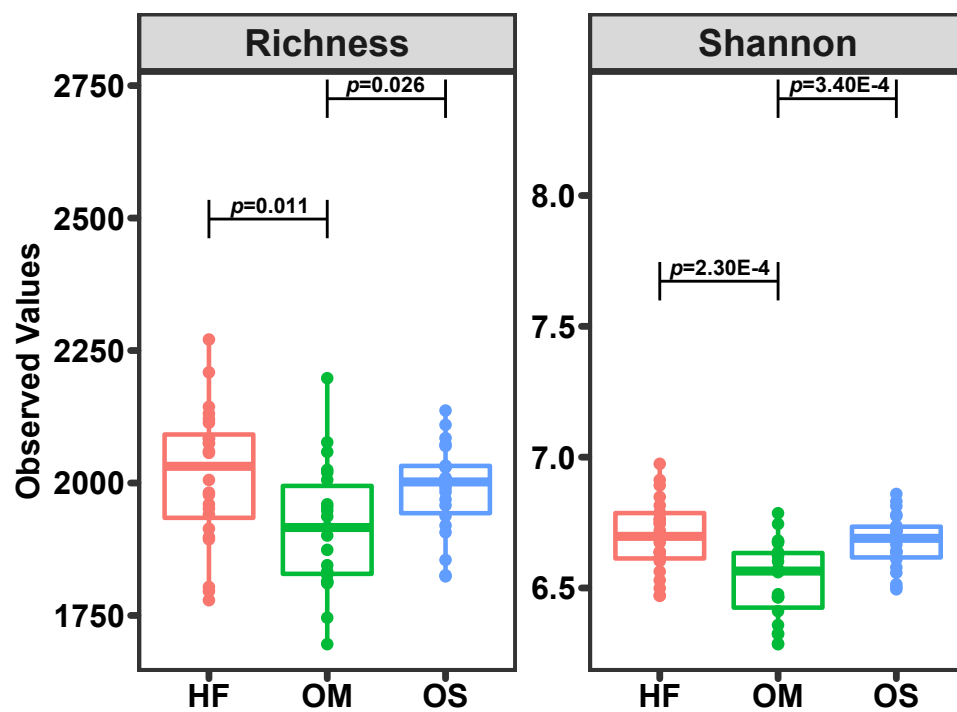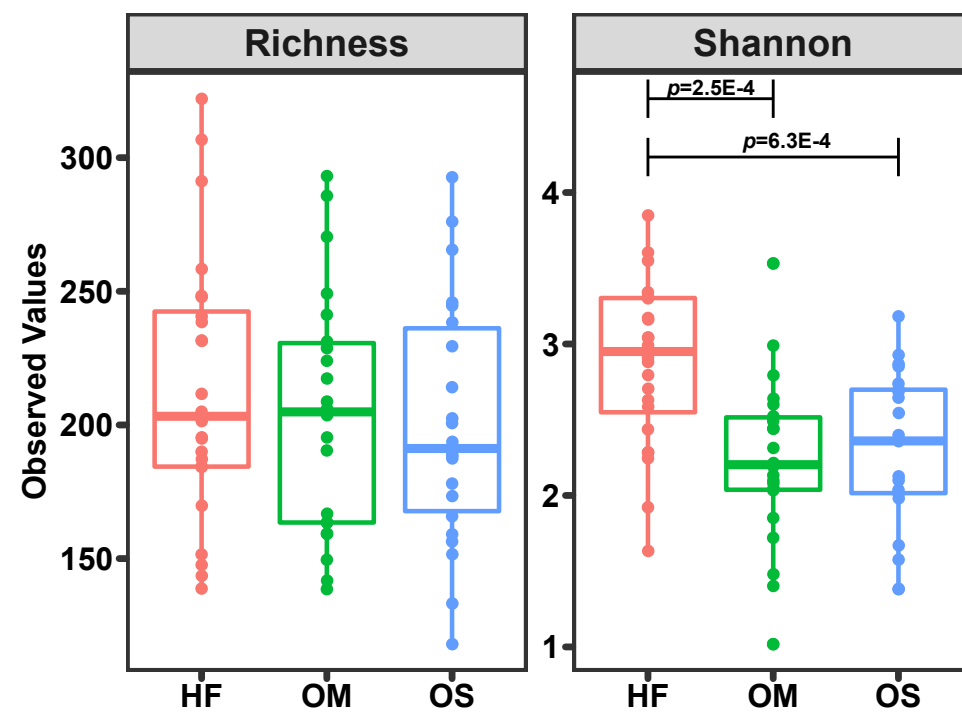

2020Dry

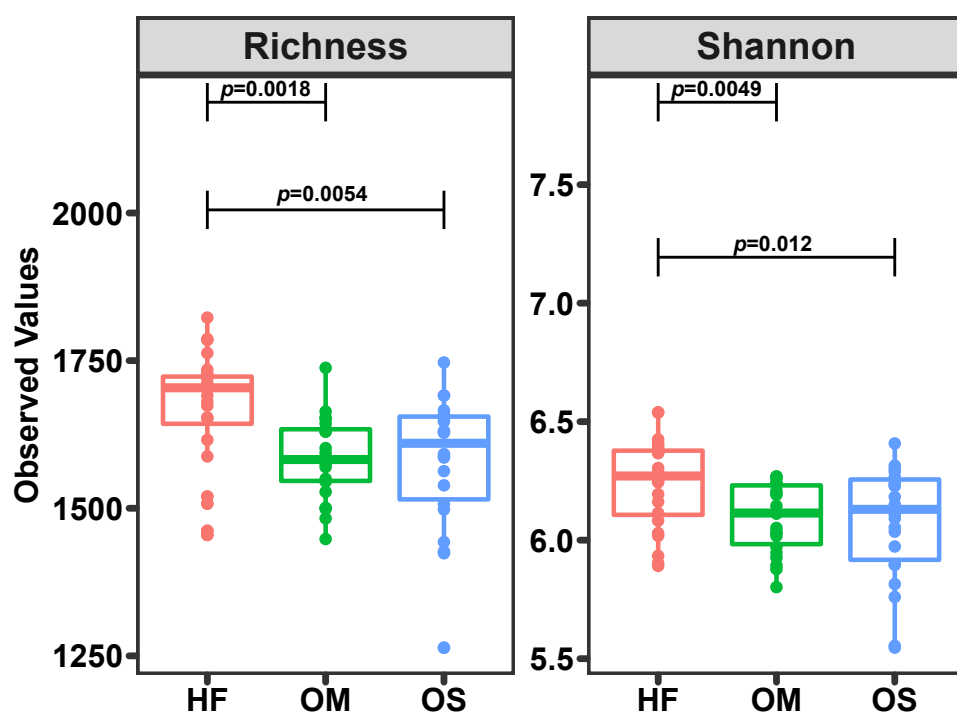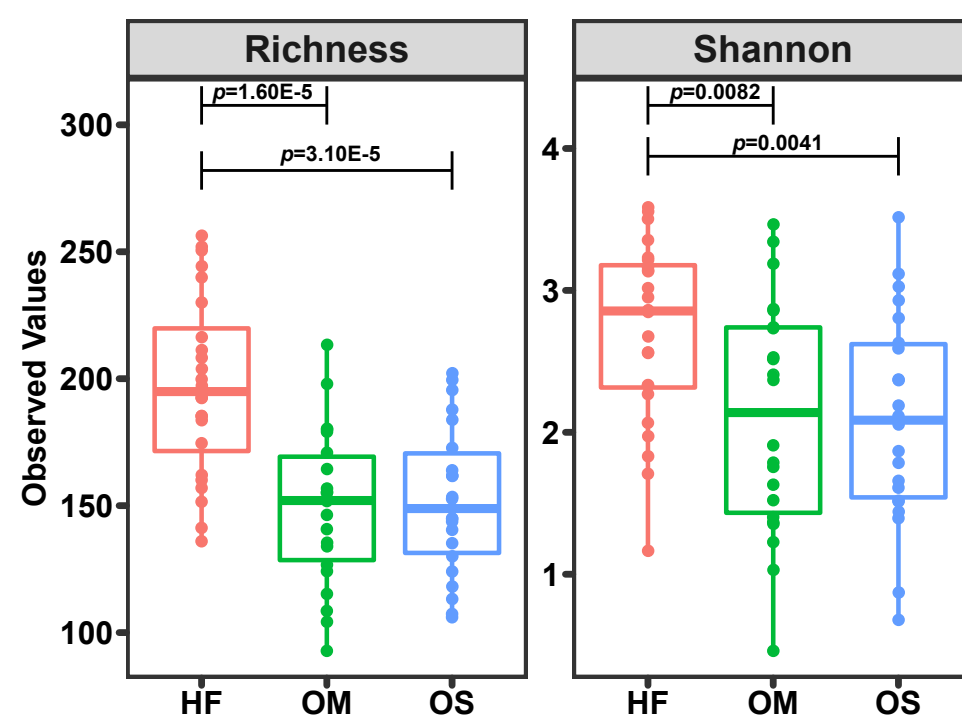

2020Wet

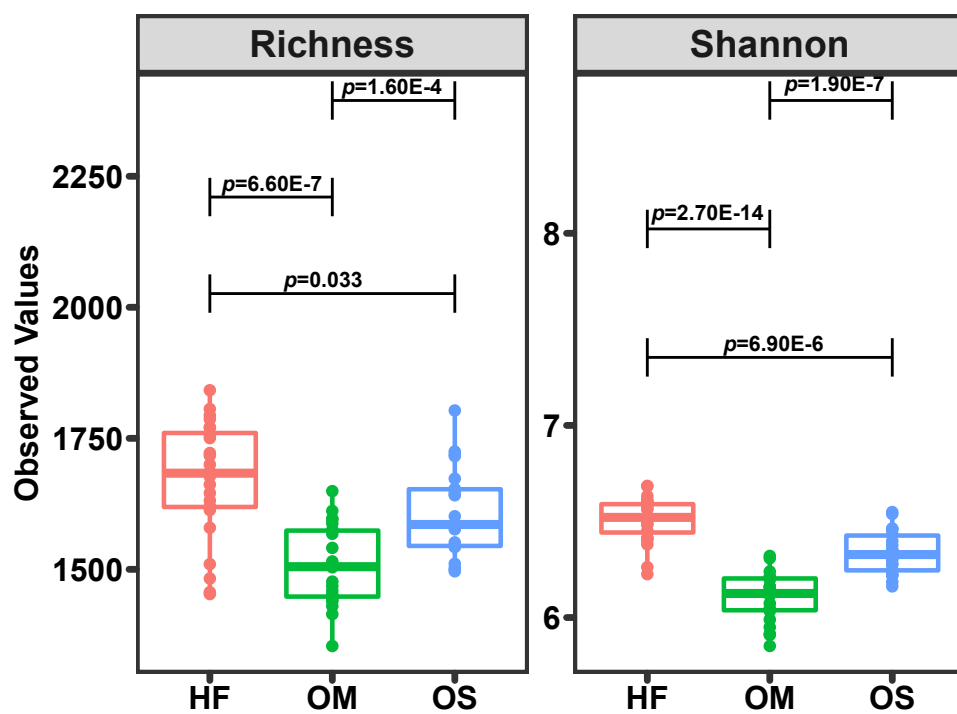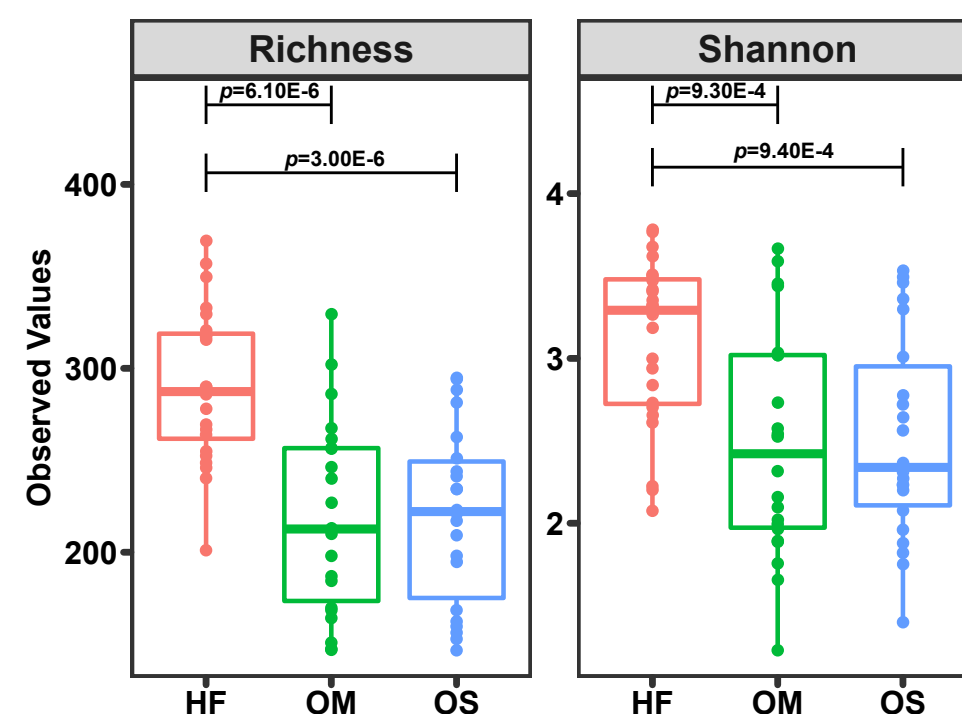

Supplement: Supplementary file 1 [file Data_Sheet_1.zip › Supplementary files/Figure S2.pdf]

**A**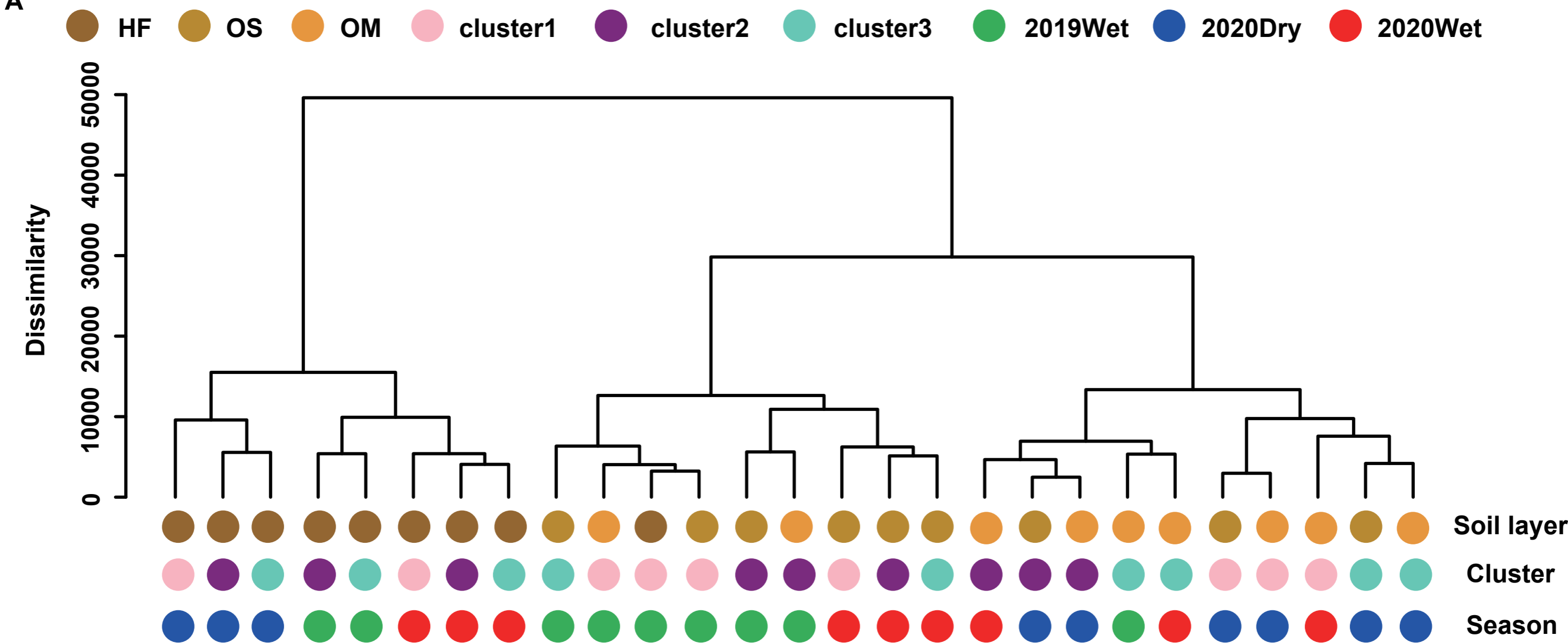**B**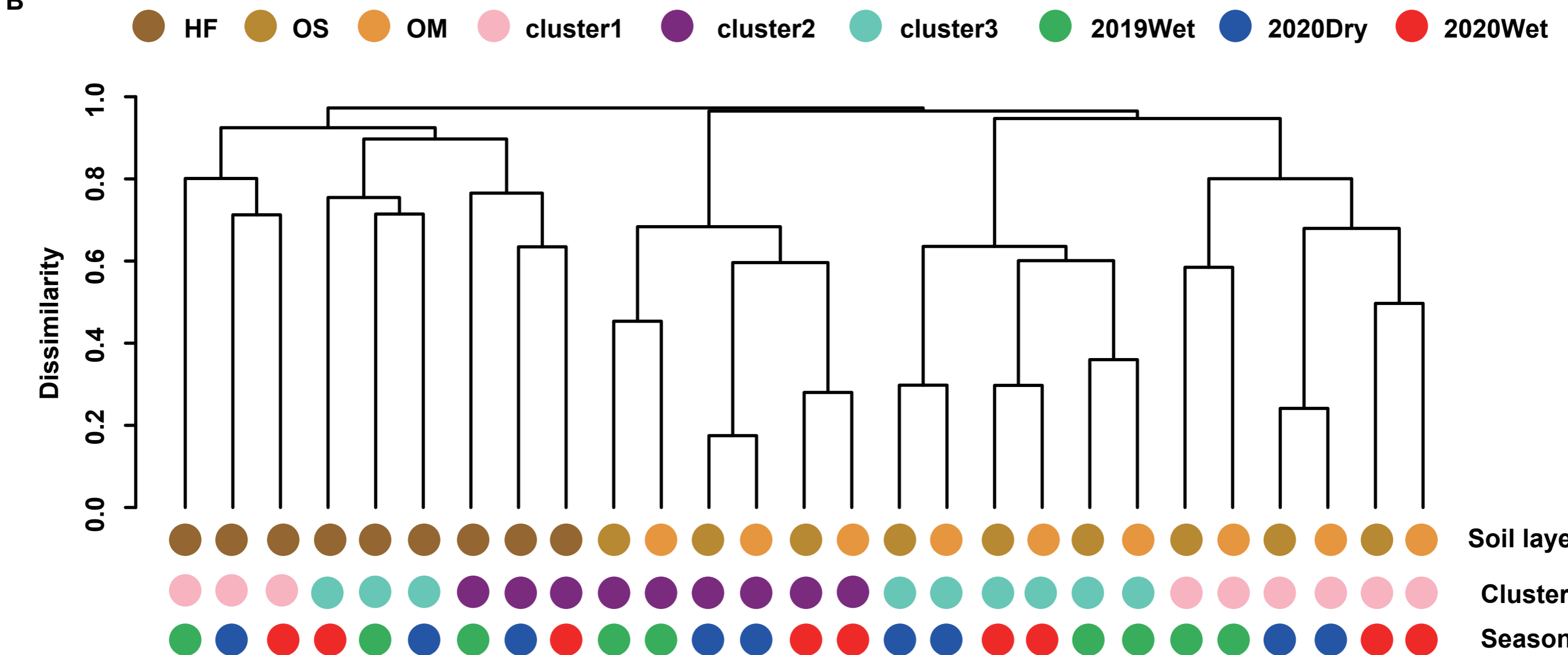

Supplement: Supplementary file 1 [file Data_Sheet_1.zip › Supplementary files/Figure S3.pdf]

● 2019Wet ● 2020Dry ● 2020Wet

HF

OS

OM

Bacteria

NMDS2

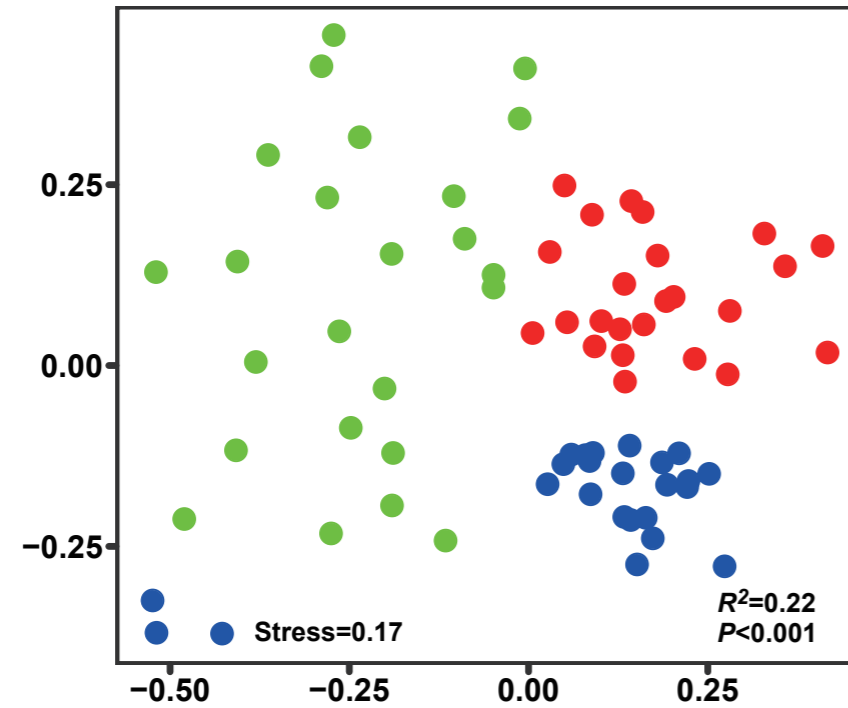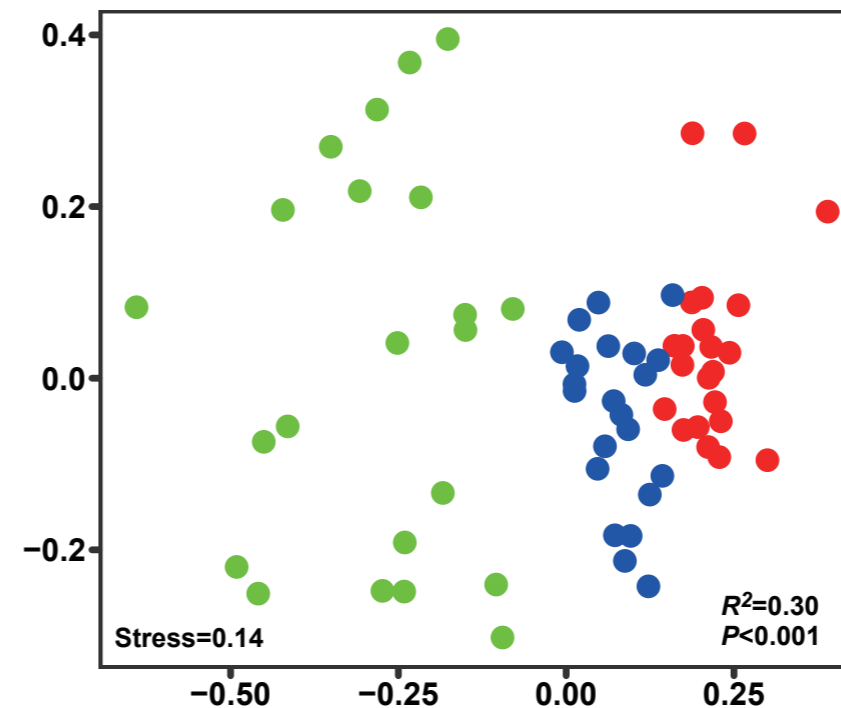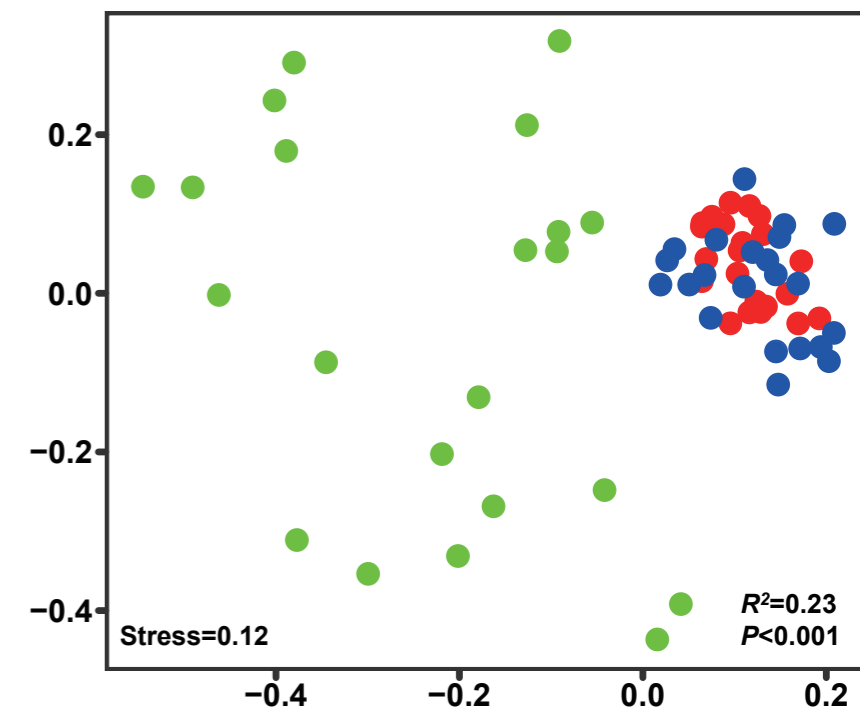

Fungi

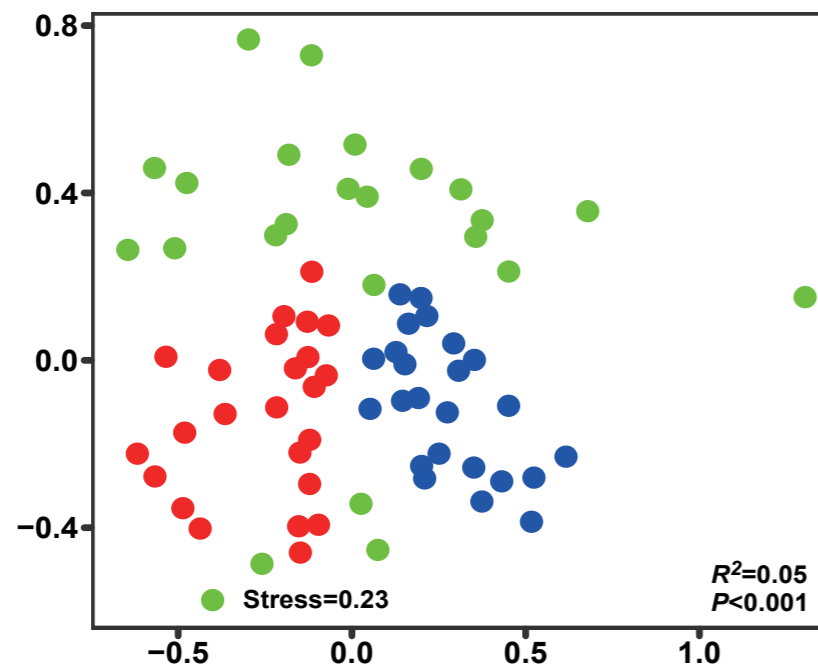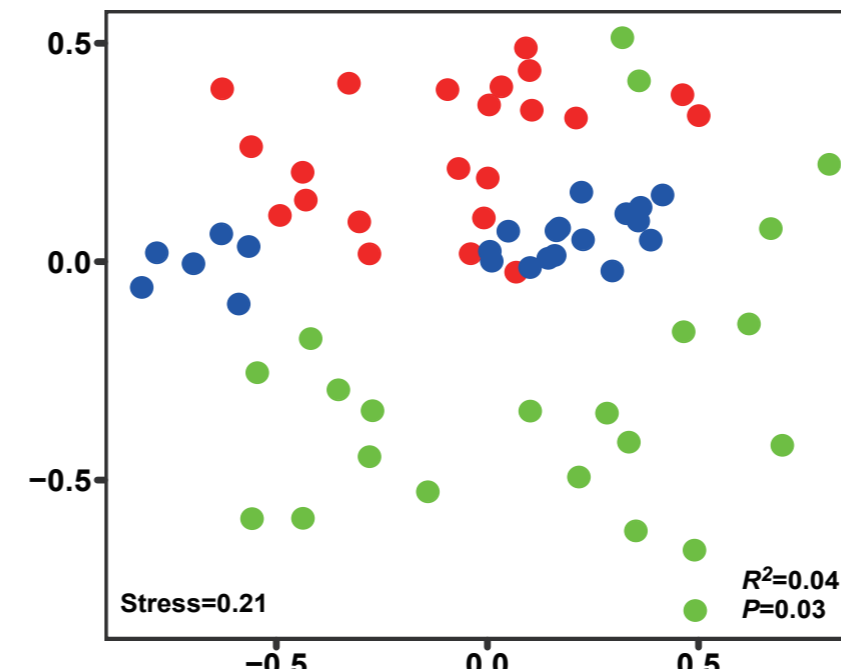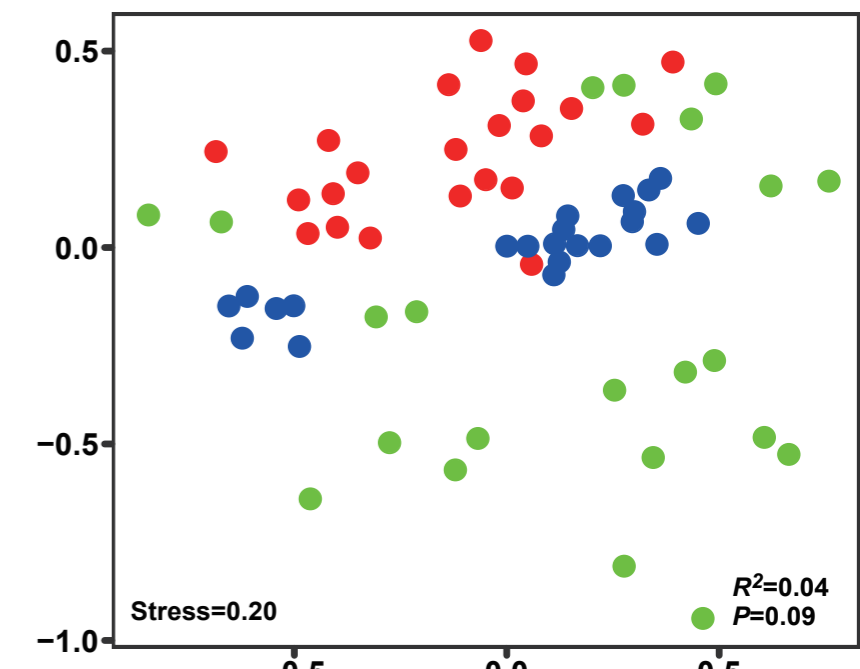

NMDS1

Supplement: Supplementary file 1 [file Data_Sheet_1.zip › Supplementary files/Figure S4.pdf]
