# Supplementary material for: Impact of the soil layer on the soil microbial diversity and composition of Pinus yunnanensis at the Ailao Mountains subtropical forest
Source: Front Microbiol. 2025 May 29;16:1558906. doi: 10.3389/fmicb.2025.1558906 (PMC12159057; doi:10.3389/fmicb.2025.1558906)
Supplement: Supplementary file 1 [file Figure S16.pdf › Supplementary files/Figure S16.pdf]

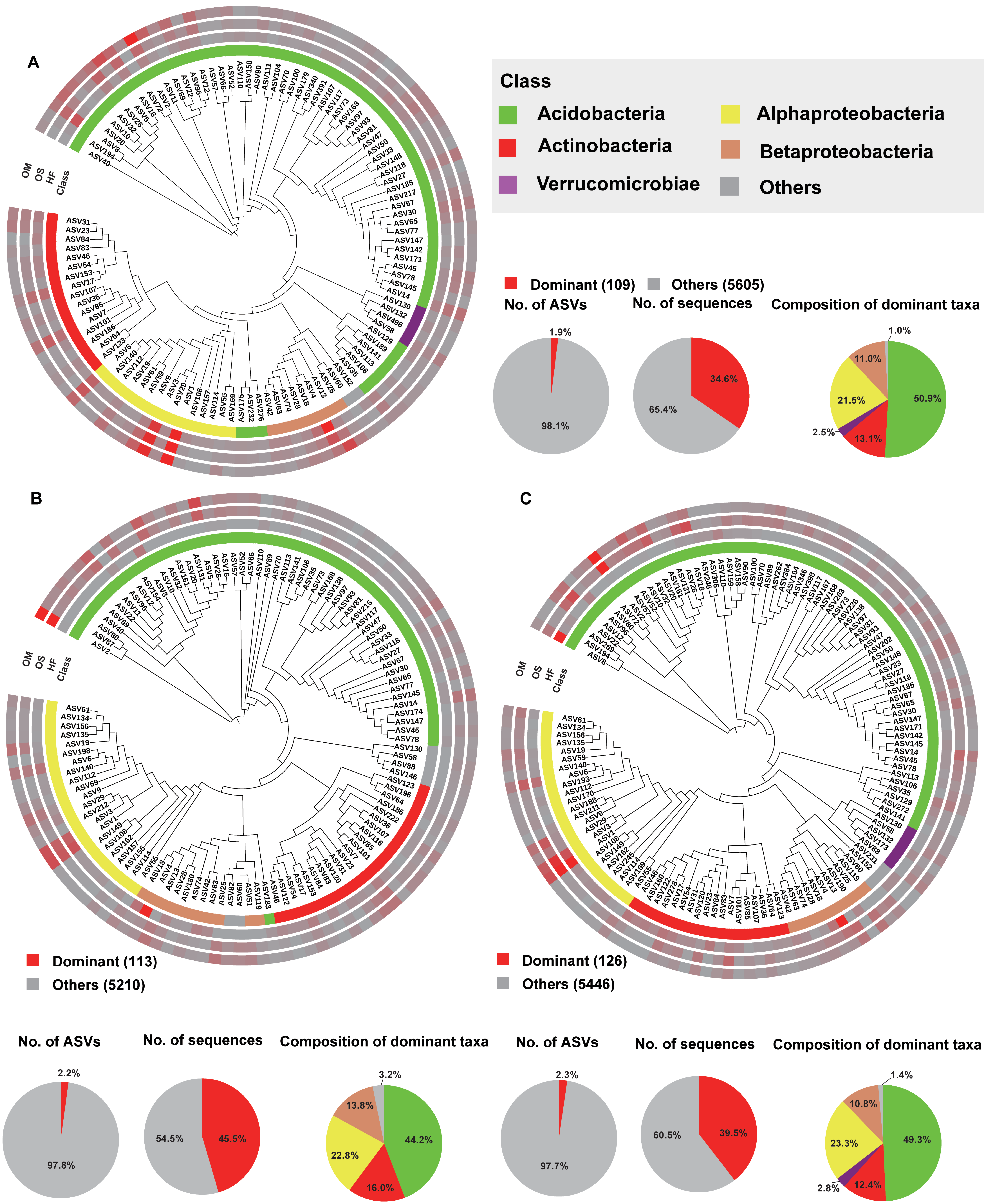
A
Class
Acidobacteria
Actinobacteria
Verrucomicrobiae
Alphaproteobacteria
Betaproteobacteria
Others
OM
OS
HF
Class
Dominant (109)
Others (5605)
No. of ASVs
No. of sequences
Composition of dominant taxa
1.9%
98.1%
34.6%
65.4%
1.0%
11.0%
21.5%
50.9%
2.5%
13.1%
B
OM
OS
HF
Class
Dominant (113)
Others (5210)
No. of ASVs
No. of sequences
Composition of dominant taxa
2.2%
97.8%
54.5%
45.5%
3.2%
13.8%
22.8%
44.2%
16.0%
C
OM
OS
HF
Class
Dominant (126)
Others (5446)
No. of ASVs
No. of sequences
Composition of dominant taxa
2.3%
97.7%
60.5%
39.5%
1.4%
10.8%
23.3%
49.3%
2.8%
12.4%
