# Supplementary material for: Impact of the soil layer on the soil microbial diversity and composition of Pinus yunnanensis at the Ailao Mountains subtropical forest
Source: Front Microbiol. 2025 May 29;16:1558906. doi: 10.3389/fmicb.2025.1558906 (PMC12159057; doi:10.3389/fmicb.2025.1558906)
Supplement: Supplementary file 1 [file Figure S17.pdf › Supplementary files/Figure S17.pdf]

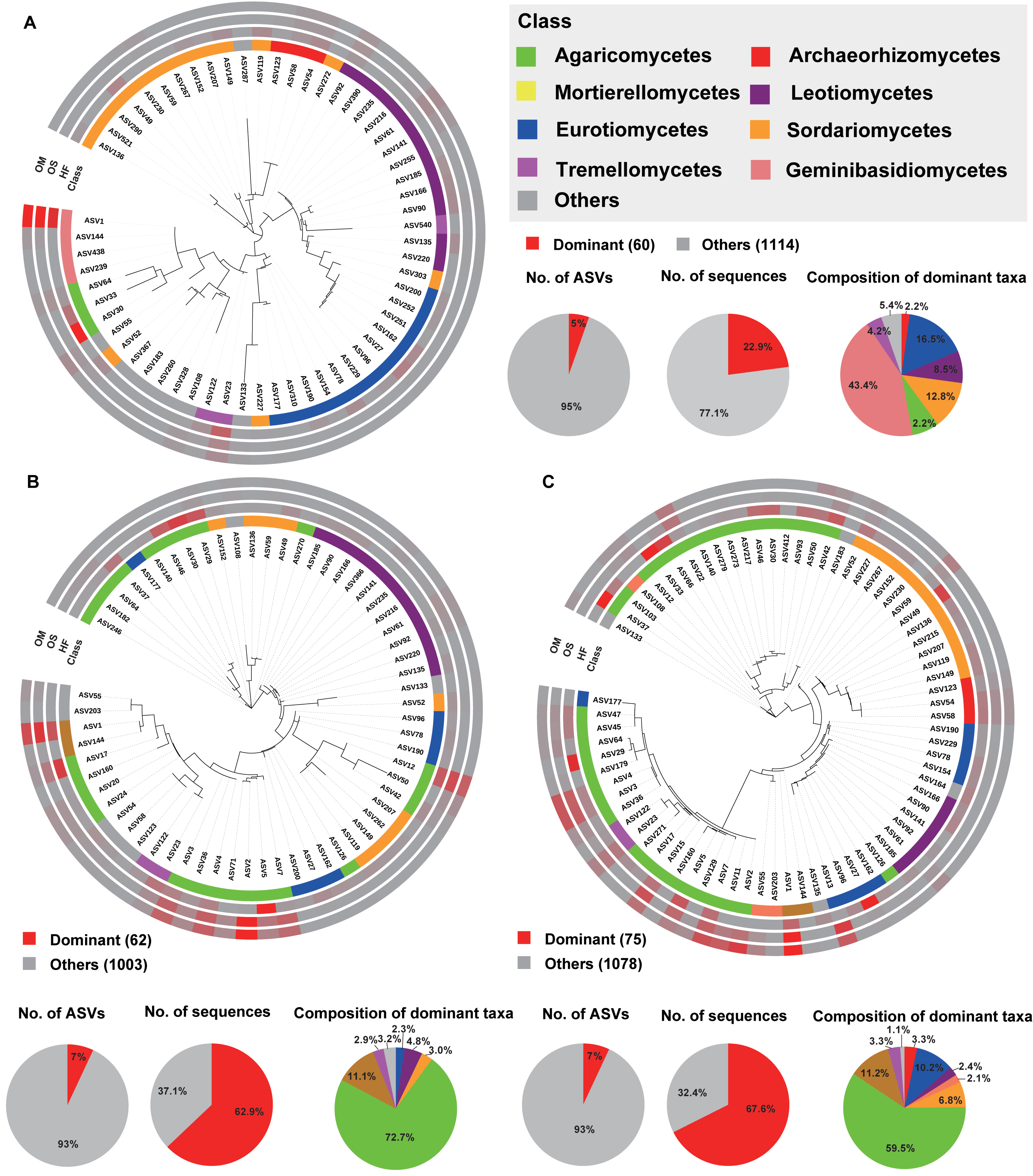
A
Class
Agaricomycetes
Archaeorhizomycetes
Mortierellomycetes
Leotiomycetes
Eurotiomycetes
Sordariomycetes
Tremellomycetes
Geminibasidiomycetes
Others
OM
OS
HF
Class
ASV136 ASV521 ASV290 ASV49 ASV230 ASV59 ASV267 ASV152 ASV207 ASV149 ASV287 ASV119 ASV123 ASV58 ASV54 ASV272 ASV92 ASV390 ASV235 ASV216 ASV61 ASV141 ASV255 ASV185 ASV166 ASV90 ASV540 ASV135 ASV220 ASV303 ASV200 ASV252 ASV251 ASV162 ASV27 ASV96 ASV229 ASV78 ASV154 ASV190 ASV310 ASV177 ASV227 ASV133 ASV23 ASV122 ASV108 ASV328 ASV260 ASV183 ASV367 ASV52 ASV55 ASV30 ASV33 ASV64 ASV239 ASV438 ASV144 ASV1
Dominant (60)
Others (1114)
No. of ASVs
5%
95%
No. of sequences
22.9%
77.1%
Composition of dominant taxa
5.4%
2.2%
16.5%
8.5%
12.8%
2.2%
43.4%
4.2%
B
OM
OS
HF
Class
ASV246 ASV182 ASV64 ASV37 ASV177 ASV140 ASV46 ASV30 ASV29 ASV152 ASV108 ASV136 ASV59 ASV49 ASV270 ASV185 ASV90 ASV166 ASV366 ASV141 ASV235 ASV216 ASV61 ASV92 ASV220 ASV135 ASV133 ASV52 ASV96 ASV78 ASV190 ASV12 ASV50 ASV42 ASV207 ASV262 ASV149 ASV119 ASV126 ASV162 ASV27 ASV200 ASV7 ASV5 ASV2 ASV71 ASV4 ASV36 ASV3 ASV23 ASV122 ASV123 ASV58 ASV54 ASV24 ASV20 ASV160 ASV17 ASV144 ASV1 ASV203 ASV55
Dominant (62)
Others (1003)
No. of ASVs
7%
93%
No. of sequences
37.1%
62.9%
Composition of dominant taxa
2.3%
2.9%
3.2%
4.8%
3.0%
11.1%
72.7%
C
OM
OS
HF
Class
ASV133 ASV37 ASV103 ASV108 ASV12 ASV33 ASV66 ASV22 ASV140 ASV279 ASV273 ASV217 ASV46 ASV30 ASV412 ASV93 ASV50 ASV42 ASV183 ASV52 ASV227 ASV267 ASV152 ASV230 ASV59 ASV49 ASV136 ASV215 ASV207 ASV119 ASV149 ASV123 ASV54 ASV58 ASV190 ASV229 ASV78 ASV154 ASV164 ASV166 ASV90 ASV141 ASV92 ASV61 ASV185 ASV126 ASV162 ASV27 ASV96 ASV13 ASV135 ASV144 ASV1 ASV203 ASV55 ASV2 ASV11 ASV7 ASV129 ASV5 ASV160 ASV15 ASV17 ASV271 ASV23 ASV122 ASV36 ASV3 ASV4 ASV179 ASV29 ASV64 ASV45 ASV47 ASV177
Dominant (75)
Others (1078)
No. of ASVs
7%
93%
No. of sequences
32.4%
67.6%
Composition of dominant taxa
1.1%
3.3%
3.3%
10.2%
2.4%
2.1%
11.2%
6.8%
59.5%
